# Genetic and environmental variation impact the cuticular hydrocarbon metabolome on the stigmatic surfaces of maize

**DOI:** 10.1186/s12870-019-2040-3

**Published:** 2019-10-17

**Authors:** Tesia Dennison, Wenmin Qin, Derek M. Loneman, Samson G. F. Condon, Nick Lauter, Basil J. Nikolau, Marna D. Yandeau-Nelson

**Affiliations:** 1 Interdepartmental Genetics and Genomics Graduate Program, Iowa State University, Ames, USA; 2 Department of Plant Pathology and Microbiology, Iowa State University, Ames, USA; 3 Roy J. Carver Department of Biochemistry, Biophysics, and Molecular Biology, Iowa State University, Ames, USA; 4 Present Address: GenScript, Nanjing, China; 5 Department of Genetics, Development, and Cell Biology, Iowa State University, Ames, USA; 6 Present Address: School of Medicine, Case Western Reserve University, Cleveland, OH USA; 7 Present Address: Department of Biochemistry, University of Wisconsin, Madison, USA; 8 USDA-ARS Corn Insects and Crop Genetics Research Unit, Iowa State University, Ames, USA; 9 NSF-Engineering Research Center for Biorenewable Chemicals, Iowa State University, Ames, USA; 10 Center for Metabolic Biology, Iowa State University, Ames, USA

**Keywords:** Maize silks, Cuticle, Cuticular wax, Surface lipids, Fatty acid synthesis, Desaturation, Hydrocarbons, Metabolomics, NAM founders

## Abstract

**Background:**

Simple non-isoprenoid hydrocarbons accumulate in discrete regions of the biosphere, including within bacteria and algae as a carbon and/or energy store, and the cuticles of plants and insects, where they may protect against environmental stresses. The extracellular cuticular surfaces of the stigmatic silks of maize are rich in linear hydrocarbons and therefore provide a convenient system to study the biological origins and functions of these unique metabolites.

**Results:**

To test the hypotheses that genetics and environment influence the accumulation of surface hydrocarbons on silks and to examine the breadth of metabolome compositions across diverse germplasm, cuticular hydrocarbons were analyzed on husk-encased silks and silks that emerged from the husk leaves from 32 genetically diverse maize inbred lines, most of which are commonly utilized in genetics experiments. Total hydrocarbon accumulation varied ~ 10-fold among inbred lines, and up to 5-fold between emerged and husk-encased silks. Alkenes accounted for 5-60% of the total hydrocarbon metabolome, and the majority of alkenes were monoenes with a double bond at either the 7th or 9th carbon atom of the alkyl chain. Total hydrocarbon accumulation was impacted to similar degrees by genotype and husk encasement status, whereas genotype predominantly impacted alkene composition. Only minor differences in the metabolome were observed on silks that were emerged into the external environment for 3- versus 6-days. The environmental influence on the metabolome was further investigated by growing inbred lines in 2 years, one of which was warmer and wetter. Inbred lines grown in the drier year accumulated up to 2-fold more hydrocarbons and up to a 22% higher relative abundance of alkenes. In summary, the surface hydrocarbon metabolome of silks is primarily governed by genotype and husk encasement status, with smaller impacts of environment and genotype-by-environment interactions.

**Conclusions:**

This study reveals that the composition of the cuticular hydrocarbon metabolome on silks is affected significantly by genetic factors, and is therefore amenable to dissection using quantitative genetic approaches. Such studies will clarify the genetic mechanisms responsible for the accumulation of these metabolites, enabling detailed functional investigations of the diverse and complex protective roles of silk surface lipids against environmental stresses.

## Background

The plant cuticle is the outermost physical barrier between most aerial portions of plants and the external environment. The cuticle is produced and secreted by epidermal cells and consists of a polyester cutin matrix, which is embedded with and coated by a complex mixture of unique, and readily extractable, non-polar extracellular cuticular surface lipids [1, 2]. These surface lipid metabolites, also commonly referred to as cuticular waxes or cuticular lipids, can include very-long chain fatty acids (VLCFAs) of 20 carbons or greater and derivatives of these VLCFAs, including fatty aldehydes, primary and secondary alcohols, wax esters, and hydrocarbons. The presence and relative compositions of these lipid classes vary among organisms, and can also vary between plant tissues and developmental stages of individual tissues and organs [2, 3]. Functionally, cuticular surface lipids provide a hydrophobic barrier that limits transpirational water loss and protects against abiotic and biotic stresses, such as drought, frost, ultraviolet radiation, pests and pathogens [1, 4–7].

Simple, straight-chain hydrocarbons are predominant constituents within the cuticles of insects [8] and the cuticular surface lipids of many plants [9–14]. Hydrocarbons comprise more than 50% of surface lipid constituents in specific tissues of plants, such as leaves, flowers and siliques of Arabidopsis; stems of alfalfa; flowers of camelina; and leaves of rapeseed and tomato [2]. In maize, although hydrocarbons are minor surface lipid constituents of juvenile leaves (1%) [15] and kernels (6%) [16], they are present at higher levels on adult leaves (17%) [16] and pollen (15-50%) [17]. Silks are particularly rich in hydrocarbons, comprising 40-90% of the silk surface lipid metabolome in specific inbred lines [18–20].

Maize silks are the stigmatic portions of the female flowers that provide the conduits for fertilization by pollen. During the critical period of pollination, silks that have emerged from the encasing husk leaves are particularly vulnerable to abiotic and biotic stresses, and cuticular surface lipids are hypothesized to provide the first line of defense. Surface lipid accumulation on maize silks has previously been profiled from the agronomically important inbred lines, B73 and Mo17 [18, 19], and germplasm selected for resistance or susceptibility to biological stresses (e.g. corn earworm and pathogenic fungi) [20, 21]. In support of the protective role of surface lipid metabolites, hydrocarbons accumulate to 2- to 5-fold higher levels on the portions of the silks that have emerged from the encasing husk leaves into the external environment as compared to the portions of the silks that are encased by the protective husk leaves for the small numbers of inbred lines that have been examined [18, 19, 21]. Also, cuticular surface hydrocarbon accumulation has been shown to be impacted by the length of time the silks are exposed to the external environment [18, 19, 21]. For example, hydrocarbon content on B73 silks was shown to increase from 0.2 to 1.2% of silk dry weight from the first to the seventh day post-silk emergence (PSE), with a drastic increase between 2- and 3-days PSE [19].

Although the protective capacities of hydrocarbons within the plant cuticle are not fully elucidated, evidence suggests roles against both biotic and abiotic stresses in maize and other organisms. Cuticular hydrocarbons on maize silks are suggested to be associated with reducing insect herbivory; in particular, silk feeding by corn ear worms (*Helicoverpa zea* [Boddie]). Specifically, corn ear worm larval growth was inhibited by diets containing organically extracted cuticular lipids from silks of particular maize genotypes [20]. Similarly, cuticular hydrocarbons have been shown to impact egg-laying behaviors (ovipositioning) of European corn borer on adult maize leaves [22]. Evidence also suggests that hydrocarbons provide a cuticular water and vapor barrier, especially under drought conditions. For example, water deficit conditions resulted in increased cuticular hydrocarbons, specifically alkane accumulation, in soybean [23], sesame [24], cotton [25], and Arabidopsis [26]. In Arabidopsis, it has been demonstrated that water deficit induced the expression of the hydrocarbon biosynthesis gene, *ECERIFERUM* (*CER1*), with a concomitant increase in alkane concentration as well as decreased rates of water loss (i.e. cuticular permeability) [26].

The breadth of genetic diversity available in maize has not yet been utilized for silk surface lipid metabolome investigations. To test the hypothesis that genetics and environment significantly affect the accumulation of surface hydrocarbons on maize silks, we evaluated the breadth of metabolome compositions for 32 genetically diverse maize inbred lines, including founders of the Nested Association Mapping (NAM) population [27], which provides the future ability to genetically dissect these traits either in a NAM or other genetic mapping framework. We profiled hydrocarbon metabolites on emerged and husk-encased silks from each of these inbred lines to assess the impacts of both genotype and silk emergence from the husk leaves on the composition of the surface hydrocarbon metabolome. Additionally, subsets of the panel were investigated at both 3- and 6-days PSE (15 inbred lines) and in two growing years (7 inbred lines) to evaluate the influence of increased environmental exposure on this metabolome and to investigate interactions between genotype and the environment (i.e. *GxE* interactions).

## Results

In this study, we surveyed cuticular surface hydrocarbon accumulation on silks from 32 genetically diverse maize inbred lines, including nine inbred lines selected from previously identified groupings of maize inbred lines that harbor high genome-wide diversity [28], four “expired-plant variety protection” (ex-PVP) commercial inbred lines, and 19 of the genetically diverse founder inbred lines of the maize NAM population [27] (Table 1). To assess the impact of both genetics and environment on the surface hydrocarbon metabolome, we evaluated surface hydrocarbon composition on 1) emerged versus husk-encased silks, which are exposed to different micro-environments; 2) silks that differed in the duration of exposure to the external environment using silks collected 3- versus 6-days PSE; and 3) silks from inbred lines grown in two consecutive years to assess the impacts of environmental differences between growing years and *GxE* interactions. For the studies conducted at 3-days PSE, the 2010 dataset that includes 22 inbred lines, most of which are NAM founders and perhaps of broadest interest, is presented first followed by the 2009 dataset that includes 16 inbred lines. Gas chromatography-mass spectrometry (GC-MS) metabolite profiling of hydrocarbon extracts revealed a set of 28 saturated and unsaturated hydrocarbons (i.e. alkanes and alkenes, respectively), ranging in acyl chain lengths of 21 to 31 carbon atoms (Table 2). Additional file 1: Table S1 reports concentrations of each individual constituent (μmole/g dry weight of silks), which varied in total accumulation and relative composition depending on genotype and environment parameters.

**Table 1:** Maize inbred lines surveyed for compositional variation in the silk surface hydrocarbon metabolome. NAM founder [27] and expired-Plant Variety Protection (ex-PVP) inbred lines are indicated with carats and asterisks, respectively. Heterotic group and heterotic subgroup are indicated as defined by Liu et al. [28] and Mikel and Dudley [29], with the following abbreviations: SS, stiff stalk; NSS, non-stiff stalk; TS, tropical or semitropical; Mixed, < 80% membership to any one heterotic group. PSE, post-silk emergence

Inbred line	Heterotic Group	Heterotic Subgroup	Sample size (n)		
			2009		2010
			3-days PSE	6-days PSE	3-days PSE
A632	SS	B14A	5	6	Not evaluated
B37	SS	B37	5	5	Not evaluated
B73^∧^	SS	B73	6	4	14
B97	NSS	NSS-mixed	Not evaluated	Not evaluated	5
C103	NSS	C103	Not evaluated	3	Not evaluated
CML228^∧^	TS	Suwan	Not evaluated	Not evaluated	5
CML277^∧^	TS	CML-P	Not evaluated	Not evaluated	5
CML322^∧^	TS	CML-early	6	Not evaluated	4
CML333^∧^	TS	CML-P	Not evaluated	Not evaluated	5
CML5	TS	CML-late	6	6	Not evaluated
CML52^∧^	TS	TZI	6	6	5
CML69^∧^	TS	Suwan	Not evaluated	Not evaluated	5
CML91	Mixed	Unknown	6	5	Not evaluated
H99	NSS	NSS-mixed	5	6	Not evaluated
IL14H^∧^	Sweet corn	Unknown	Not evaluated	Not evaluated	5
Ki11^∧^	TS	Suwan	Not evaluated	Not evaluated	5
Ki3^∧^	TS	Suwan	Not evaluated	Not evaluated	5
Ky21^∧^	NSS	K64W	Not evaluated	Not evaluated	5
LH1*	SS	Unknown	6	6	Not evaluated
LH123HT*	NSS	Unknown	5	6	Not evaluated
M37W^∧^	Mixed	Unknown	5	5	3
Mo17	NSS	C109:Mo17	6	5	11
Mo18W^∧^	Mixed	Unknown	Not evaluated	Not evaluated	5
NC350^∧^	TS	NC	Not evaluated	Not evaluated	3
NC358^∧^	TS	TZI	Not evaluated	Not evaluated	5
Oh43^∧^	NSS	M14:Oh43	6	4	Not evaluated
Oh7B^∧^	Mixed	Unknown	Not evaluated	Not evaluated	5
PHG39*	SS	Unknown	5	6	7
PHG84*	NSS	Unknown	Not evaluated	6	5
Tx303^∧^	Mixed	Unknown	Not evaluated	Not evaluated	5
Tzi18	TS	TZI	5	6	Not evaluated
Tzi8^∧^	TS	TZI	6	4	4

**Table 2:** Identified silk surface hydrocarbon constituents. Alkane and alkene constituents identified across the evaluated genotypes are listed. Alkenes with double bonds between the 7th and 8th or 9th and 10th carbon atoms of the alkyl chain are reported as “:1(7)” and “:1(9)” constituents, respectively. The “:1(> 9)” constituents represent an isomeric mix of alkenes that harbor double bonds at the 10th, 11th, 12th, 13th, or 14th position in the alkyl chain. A single diene, identified as “C29:2” was observed with unidentified double bond positions

Individual Surface Hydrocarbon Constituents	
Alkane constituents	C21, C23, C24, C25, C27, C28, C29, C30, C31
Alkene constituents	
7-Monoenes	C23:1 (7), C24:1 (7), C25:1 (7), C26:1 (7), C27:1 (7), C28:1 (7), C29:1 (7), C30:1 (7), C31:1 (7)
9-Monoenes	C23:1 (9), C24:1 (9), C25:1 (9), C27:1 (9), C29:1 (9), C30:1 (9), C31:1 (9)
> 9-Monoenes	C29:1(> 9), C31:1(> 9)
Diene	C29:2

### Genotype and silk micro-environment govern the abundance of surface hydrocarbons

**Fig. 1:**
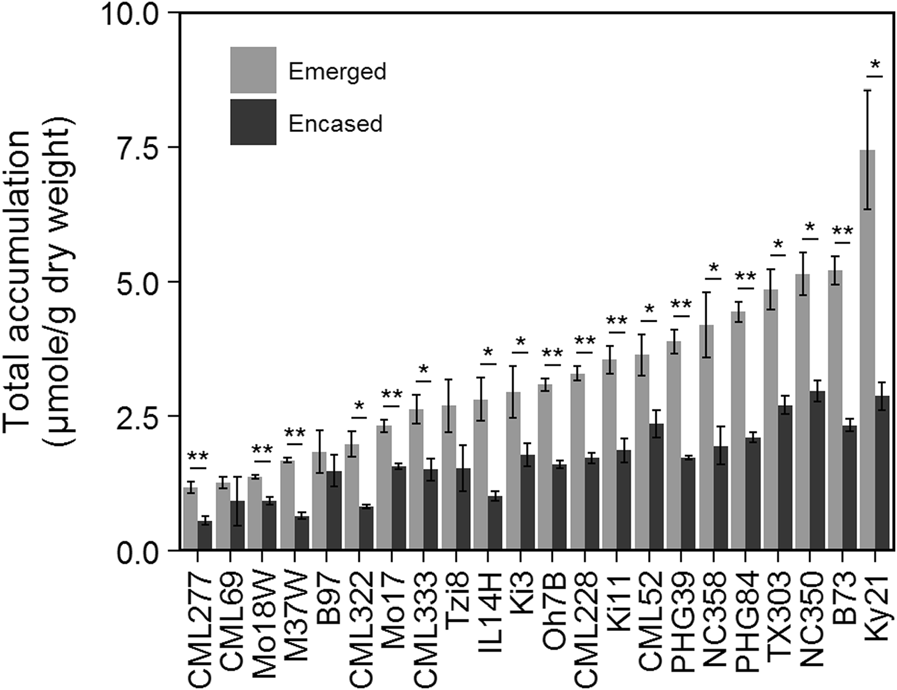
Total hydrocarbon accumulation on silks collected 3-days PSE from inbred lines grown in 2010. Inbred lines are ordered from lowest to highest hydrocarbon accumulation on emerged silks. Asterisks indicate hydrocarbon accumulation on emerged silks is significantly greater than husk-encased silks for a given inbred line (T-test; * *P* < 0.05, ** *P* < 0.001). Error bars represent ± standard error

Total hydrocarbon accumulation on both the husk-encased and the emerged portions of silks (i.e. different micro-environments) harvested at 3-days PSE varied substantially in both years of the study. Across the 22 inbred lines evaluated in 2010, total hydrocarbon accumulation varied ~ 11-fold (0.81 to 9.14 μmole/g dry weight) on emerged silks and ~ 9-fold (0.37 to 3.51 μmole/g dry weight) on husk-encased silks (Fig. 1). A slightly narrower range of variation was observed across the 16 inbred lines evaluated in 2009, with ~ 9-fold variation in total hydrocarbon accumulation on emerged silks (1.30 to 11.87 μmole/g dry weight) and ~ 6-fold variation for husk-encased silks (0.80 to 4.55 μmole/g dry weight) (Additional file 2: Figure S1). Within any single inbred line, total hydrocarbon accumulation varied consistently, and often dramatically, according to silk encasement status (i.e. emerged versus husk-encased silks). Nineteen of the 22 inbred lines evaluated in 2010 showed 1.4- to 2.8-fold higher hydrocarbon levels on emerged as compared to husk-encased silks (Fig. 1), whereas a larger breadth of variation was observed in 2009, with 14 of 16 inbred lines exhibiting 1.4- to 4.8-fold higher accumulation on emerged as compared to husk-encased silks (Additional file 2: Figure S1).

Analysis of variance (ANOVA) revealed that genotype, silk encasement status, and the interaction of these factors explain 84% of the observed variance in 2010 (Additional file 3: Table S2), with genotype (partial *R*^2^ = 0.46) having more of an impact than silk encasement status (partial *R*^2^ = 0.23). In contrast, silk encasement status explains more of the variation in total hydrocarbon accumulation (partial *R*^2^ = 0.37) than genotype (partial *R*^2^ = 0.28) in the set of inbred lines grown in 2009 and analyzed at 3-days PSE. In both years, there is a significant, albeit minor, interaction of these factors (partial *R*^2^ = 0.10) (Additional file 3: Table S2), suggesting that the effect of silk emergence from the husk leaves on total hydrocarbon accumulation varies across inbred lines.

**Fig. 2:**
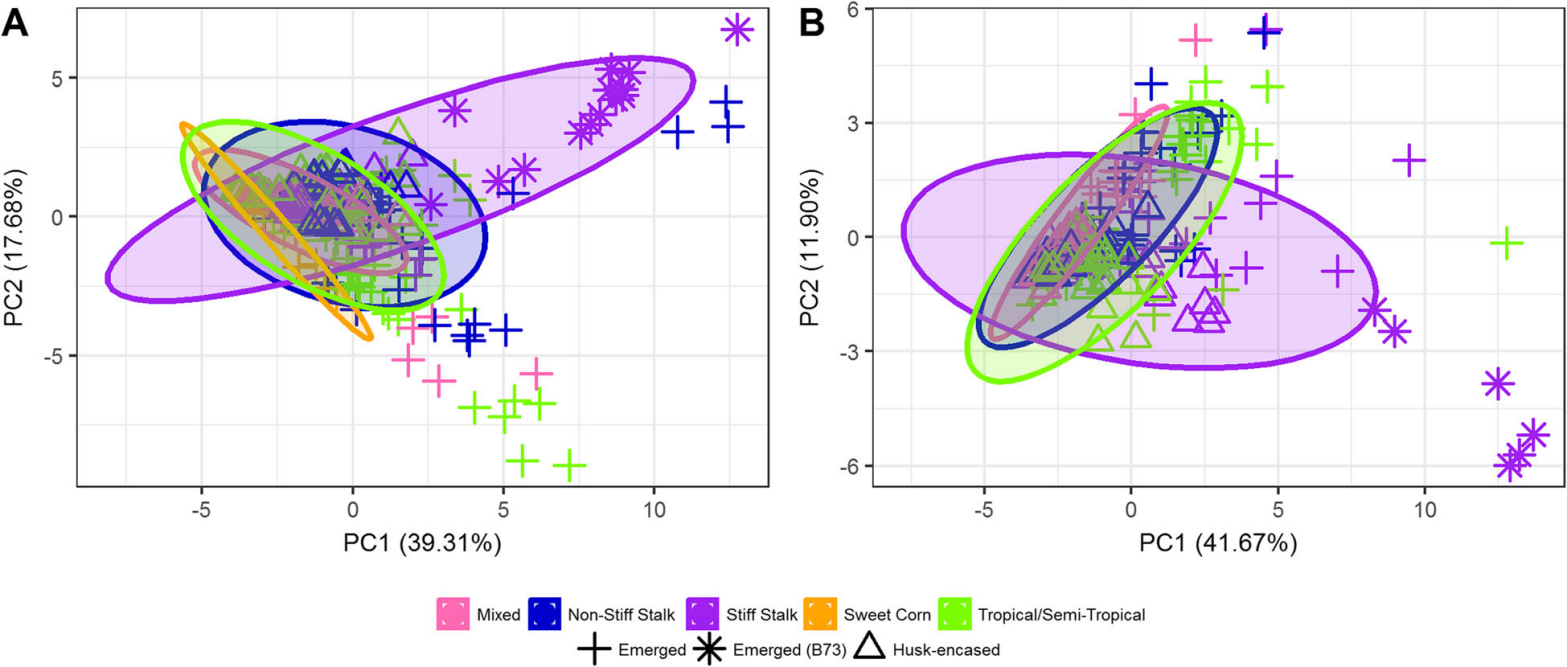
Principal component analysis of hydrocarbon constituents on emerged and husk-encased silks across five heterotic groups. The first and second principal components (PC1 and PC2) are shown for silks analyzed at 3-days PSE from the 22 and 16 inbred lines grown in 2010 (**a**) and 2009 (**b**), respectively. Percentages represent the amount of variation explained by each principal component. Ellipses represent a 95% confidence ellipse for each heterotic group. No representatives from the Sweet Corn group were grown in 2009

To explore patterns of metabolome composition across this genetically diverse set of inbred lines, we employed principal component analysis (PCA) to test the degree to which variation among surface hydrocarbon metabolomes may be associated with the five heterotic groups represented in this study (Table 1). In both the 2009 and 2010 datasets, PCA plots derived from abundances of all hydrocarbon constituents revealed that heterotic groups are largely not separable by metabolome composition (Fig. 2). This was especially true for husk-encased silks, which tightly clustered regardless of heterotic group. However, broader variation was observed among emerged silk samples, and in particular, B73 separated quite obviously from other members of the Stiff Stalk heterotic group in both years.

### Alkene composition is influenced by genotype and silk micro-environment

**Fig. 3:**
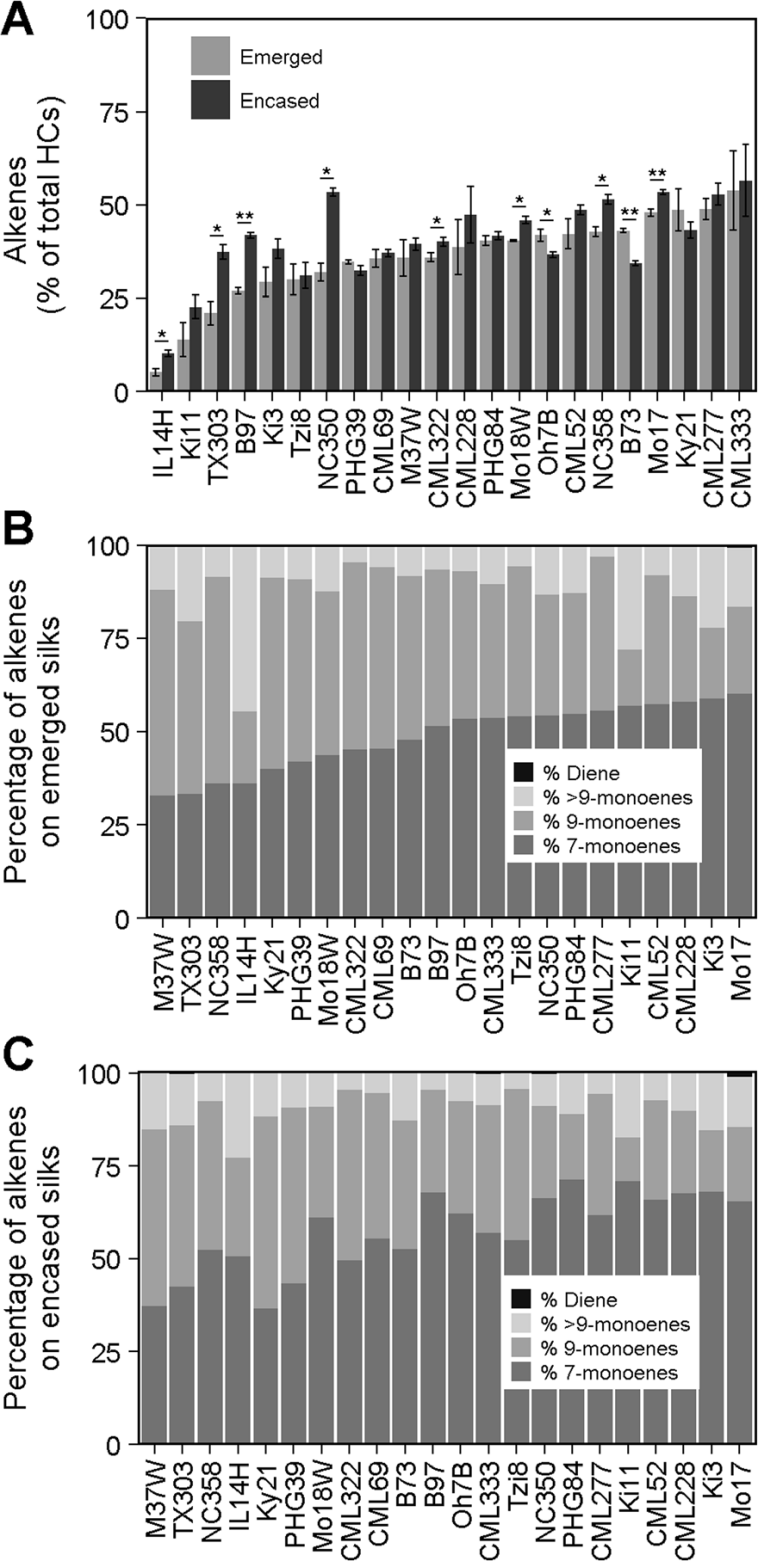
Alkene accumulation on silks collected 3-days PSE from inbred lines grown in 2010. **a** Percentage of alkenes relative to total hydrocarbons. Inbred lines are ordered by increasing percentage of alkenes on emerged silks. Asterisks indicate a significant difference between emerged and husk-encased silk means for a given inbred line (T-test; * *P* < 0.05, ** *P* < 0.001). Error bars represent ± standard error. **b** and **c** Percentage of specific alkene classes relative to total alkenes for emerged (**b**) and husk-encased (**c**) silks. Inbred lines are ordered by increasing percentage of 7-monoenes in panel (**b**) and this order is maintained in panel (**c**). HCs, hydrocarbons

The cuticular surface hydrocarbon metabolome is shown to be composed of alkanes and alkenes, primarily monoenes (i.e. single double bond in the alkyl chain), and minor amounts of dienes (i.e. two double bonds in the alkyl chain) (Additional file 1: Table S1). The relative composition of alkenes (i.e. the % of alkenes that comprise total hydrocarbons) on silks from the 22 inbred lines grown in 2010 was shown to be significantly impacted by genotype (partial *R*^2^ = 0.62), and to a much lesser extent by silk encasement status (partial *R*^2^ = 0.03) and by the interaction between these factors (partial *R*^2^ = 0.08) (Additional file 4: Table S3). The relative composition of alkenes varied widely, comprising between 5.2 and 53.9% of total hydrocarbons on emerged silks, and a similar range of variation on the husk-encased silks, 10.2 to 56.5% (Fig. 3a). For 10 of these inbred lines, there was a statistically significant difference in the relative composition of alkenes between emerged and husk-encased silks (Fig. 3a). B73 and Oh7B accumulated significantly higher percentages of alkenes on emerged silks. In contrast, eight inbred lines (IL14H, TX303, B97, NC350, CML322, Mo18W, NC358, and Mo17) accumulated higher percentages of alkenes on husk-encased as compared to emerged silks, with NC350 exhibiting the greatest difference (i.e. ~ 20%).

The silks of the 16 inbred lines grown in 2009 and analyzed at 3-days PSE showed a narrower range of relative alkene composition on emerged (24 to 49%) and husk- encased (30 to 62%) silks (Additional file 5: Figure S2), but the trends were similar to those observed with the material grown in 2010. There was a significant difference in relative alkene composition between emerged and husk-encased silks for eight of the inbred lines, with six accumulating higher percentages of alkenes on husk-encased silks (CML91, CML5, PHG39, B37, Tzi18, and Mo17). In contrast, B73 and Oh43 exhibited higher relative compositions of alkenes on emerged silks. Similar trends were observed for B73 and Mo17 in both growing years. Namely, B73 accumulated a higher percentage of alkenes on emerged silks, whereas Mo17 accumulated a higher percentage of alkenes on husk-encased silks (Fig. 3a and Additional file 5: Figure S2). Similar to the inbred lines grown in 2010, the variation observed across 2009-grown inbred lines can be explained primarily by genotype (partial *R*^2^ = 0.64), and to a much lesser extent by silk encasement and the interaction between these two factors (Additional file 4: Table S3).

To assess the influence of genotype on the double bond positions in the observed set of alkenes, we identified the double bond positions for individual alkenes after chemical derivatization with dimethyl disulfide (Additional file 6: Figure S3). Because the alkene constituent landscape was found to be comparable in the inbred lines grown in 2010 and in 2009, we report only the data for the 2010-grown material. The majority of alkenes observed in our study were monoenes that contain a double bond between the 7th and 8th carbon atoms of the alkyl chain (i.e. 7-monoenes; C23:1(7), C24:1(7), C25:1(7), C26:1(7), C27:1(7), C28:1(7), C29:1(7), C30:1(7), and C31:1(7)) or between the 9th and 10th carbon atoms (i.e. 9-monoenes; C23:1(9), C24:1(9), C25:1(9), C27:1(9), C29:1(9), C30:1(9), and C31:1(9)). Alkenes with bond positions greater than the 9th carbon in the alkyl chain were also identified, however they were not resolvable by GC-MS into separate peaks and were therefore collectively quantified as an isomeric mix designated Cn:1(> 9) (i.e. C29:1(> 9) and C31:1(> 9)). Alkenes identified in the C29:1(> 9) mixture harbor double bonds at the 10th, 11th, 12th, 13th or 14th position in the alkyl chain. The alkenes comprising the C31:1(> 9) mixture harbor identifiable double bonds at the 10th, 14th or 15th position in the alkyl chain. Although monoenes were the predominant observable class of alkenes, a single diene was also observed, C29:2. Because of the low abundance of this diene, the double bond positions were not decipherable from the dimethyl disulfide adducts.

The 7- and 9-monoenes of carbon chain lengths from 23 to 31 carbon atoms constituted the majority of the alkenes on emerged (55 to 97%; Fig. 3b) and husk-encased (77 to 96%; Fig. 3c) silks. For nine inbred lines (NC358, Mo18W, CML69, B73, B97, Oh7B, PHG84, CML277, and Mo17), we observed an increase in the percentage of 9-monoenes and concomitant decrease in the percentage of 7-monoenes for emerged silks as compared to husk-encased silks (Additional file 7: Figure S4). Similar to the relative abundance of alkenes, inbred genotype also explains the vast majority (partial *R*^2^ = 0.55) of the observed variation in the relative composition of 7- and 9-monoenes (Additional file 8: Table S4 and Additional file 9: Table S5).

### Genotypic differences in double bond position within monoenes may arise from sequence diversity within acyl-ACP desaturase genes

Variation in the relative composition of 7- and 9-monoenes suggests differences in the expression and/or regulation of fatty acid desaturation pathways among the inbred lines, and between emerged and husk-encased silks. Specifically, the observed variations in accumulation of 7- and 9-monoenes may be attributed to the differential action of acyl-acyl carrier protein (acyl-ACP) desaturases, which utilize a saturated acyl-ACP as a substrate to remove two hydrogen atoms from adjoining carbon atoms to generate a monounsaturated acyl-ACP. These acyl-ACP desaturases can have different substrate specificities (reviewed in [30]). Nucleotide polymorphisms and gene expression differences for several of these acyl-ACP desaturases have been associated with the conversion of stearic acid to oleic acid [31], suggesting that genetic diversity contributes to the observed variation in monoenes. The maize genome encodes 11 stearoyl-ACP desaturase genes (*sacd*), identified via homology to the Arabidopsis stearoyl-ACP desaturase (*AtSSI2*), which is specific for the saturated 18-carbon fatty acyl-ACP [31, 32]. We compared the protein sequences of these 11 genes in the available genome assemblies of B73 [33] and Mo17 [34] (Additional file 10: Table S6) and identified conserved catalytic diiron centers, homodimer interfaces, and putative substrate binding pockets via the National Center for Biotechnology Information Conserved Domain Database. The open reading frames (ORFs) for five of these genes were identical between these inbred lines (*sacd1, sacd2*, *sacd5*, *sacd6*, and *sacd7*). The comparison of protein sequences for *sacd10* revealed an arginine-to-histidine substitution in a conserved homodimer interface that may influence the formation of the active homodimeric form of the enzyme. The remaining five ORFs (*sacd3, sacd4, sacd8, sacd9* and *sacd11*) harbored amino acid polymorphisms outside of the recognizable functional domains, yet may still contribute to differences in protein functionality. Together, the identified polymorphisms may contribute to the variation in alkene accumulation observed herein.

### Hydrocarbons of even-numbered carbon chain lengths accumulate to low levels in a genotype-dependent manner

The prevailing biosynthesis model for the production of cuticular surface hydrocarbons focuses on fatty acid precursors of even-numbered carbon (or acyl) chain lengths (2n, where n represents the number of 2-carbon acetate units used to assemble the fatty acid) that are reduced to corresponding aldehyde intermediates, and subsequently decarbonylated to produce hydrocarbons of odd-numbered carbon chain lengths (i.e. 2n-1 carbon chain lengths) (reviewed in [35]). Indeed, the majority of saturated and unsaturated fatty acids previously observed on the surface of maize silks are of even-numbered carbon chain lengths [18–20]. In addition to these expected odd-numbered hydrocarbon constituents, hydrocarbons of even-numbered chain lengths were also shown to accumulate on the silk surfaces, albeit at low abundances (< 10% of total accumulated surface hydrocarbons; Additional file 11: Figure S5). For 18 of the 22 inbred lines grown in 2010, the relative composition of even-numbered chain length hydrocarbons significantly differed between emerged silks and their husk-encased counterparts, with emerged silks accumulating 1 to 7% more even-numbered hydrocarbons (Additional file 11: Figure S5A). Similar observations were made among the inbred lines grown in 2009 and analyzed at 3-days PSE (Additional file 11: Figure S5B). The breadth of variation in even-numbered carbon chain length hydrocarbons observed on emerged silks across the evaluated genotypes varied somewhat between the two field environments; in 2010 these constituents accounted for between 2.0 and 10.2% of the hydrocarbons among the 22 inbred lines, and this compared to 0.6 to 4.1% among the 16 inbred lines grown in 2009. The breadth of variation in husk-encased silks was considerably smaller in the two sets of inbred lines (0.5 to 1.9% in 2009 and no detectable difference to 3.4% in 2010).

The relative composition of even-numbered carbon chain length hydrocarbons was shown to be impacted to similar extents by both genotype (partial *R*^2^ = 0.36) and environmental exposure conferred by silk encasement status (partial *R*^2^ = 0.39), as well as by the interaction of these factors (partial *R*^2^ = 0.15, Additional file 12: Table S7). Hence, the environmental effect of silk encasement status on the relative abundance of even-numbered hydrocarbons is inbred-dependent. This conclusion is also supported by data gathered from inbred lines that were grown in 2009 (Additional file 12: Table S7). Collectively, these data suggest that in addition to the even-numbered carbon chain length VLCFAs that serve as precursors for hydrocarbon biosynthesis, odd-numbered VLCFAs may also serve as precursors in a decarbonylation-based pathway, albeit at a much lower frequency.

### Duration of silk exposure to the external environment affects the silk surface hydrocarbon metabolome

Because cuticular lipids have protective qualities against environmental stresses [1, 4–7], we hypothesize that silks that are exposed to the external environment for increased periods will exhibit increased levels of surface hydrocarbons as compared to silks that have been emerged for shorter periods. To assess the relative impacts of genotype, husk leaf-encasement and duration of exposure of silks to the external environment, surface hydrocarbon composition was evaluated on emerged versus husk-encased silks from 15 inbred lines grown in 2009 and evaluated at two durations of exposure to the external environment, 3- and 6-days PSE (Table 1).

**Fig. 4:**
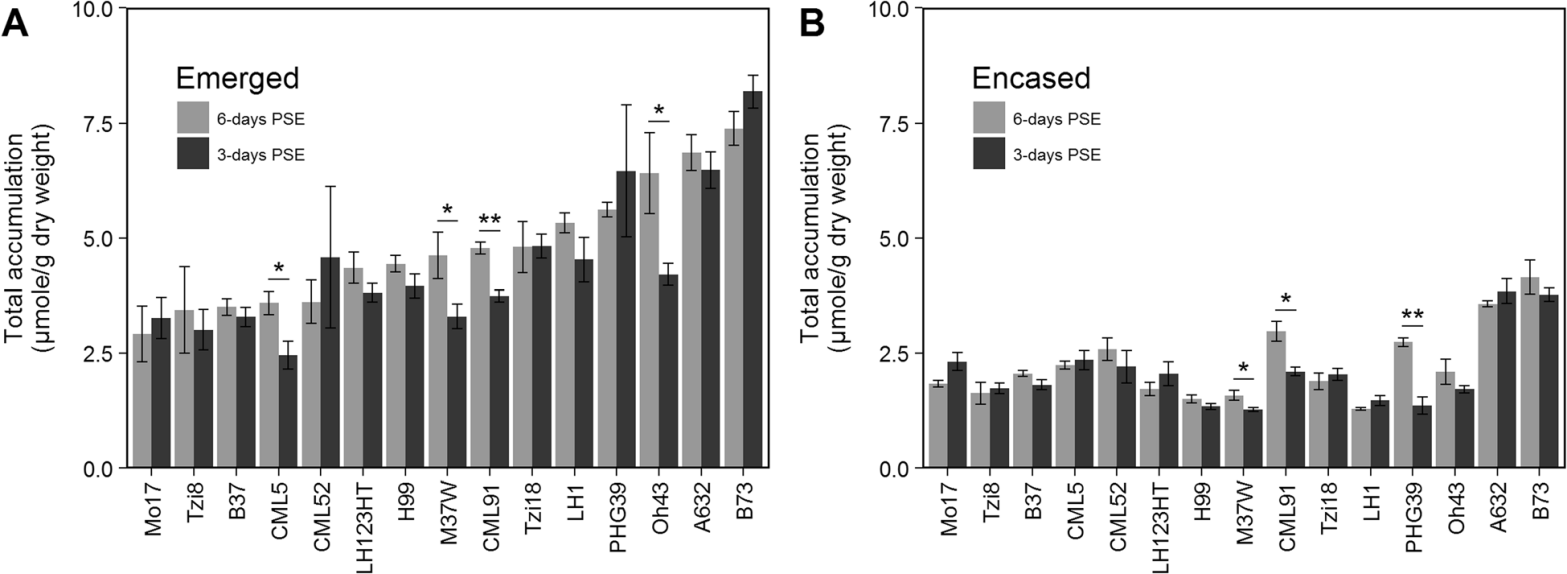
Total hydrocarbon accumulation on silks harvested at 3- and 6-days PSE. **a** and **b** Mean total hydrocarbon accumulation on emerged (**a**) and husk-encased (**b**) silks from 15 inbred lines grown in 2009. Inbred lines in panels (**a** and **b**) are ordered from low to high hydrocarbon accumulation on emerged silks at 3-days PSE (panel **a**). Asterisks indicate hydrocarbon accumulation at 6-days PSE is significantly greater than at 3-days PSE on emerged (**a**) or husk-encased (**b**) silks for a given inbred line (T-test; * *P* < 0.05, ** *P* < 0.001). Error bars represent ± standard error

Total hydrocarbon accumulation was minimally impacted by duration of exposure of silks to the external environment (partial *R*^2^ = 0.01), and as reported previously for silks evaluated at 3-days PSE (Additional file 3: Table S2), can be explained largely by silk encasement status (partial *R*^2^ = 0.49) and by genotype (partial *R*^2^ = 0.25). Even so, for four of the inbred lines, total accumulation was 1.3- to 1.5-fold higher on silks exposed to the external environment for 6-days as compared to 3-days (Fig. 4a). Interestingly, CML91 and M37W, which accumulated more hydrocarbons on emerged silks, also exhibited 1.2- to 2.0-fold higher hydrocarbon accumulation on husk-encased silks at 6-days PSE (Fig. 4b).

**Fig. 5:**
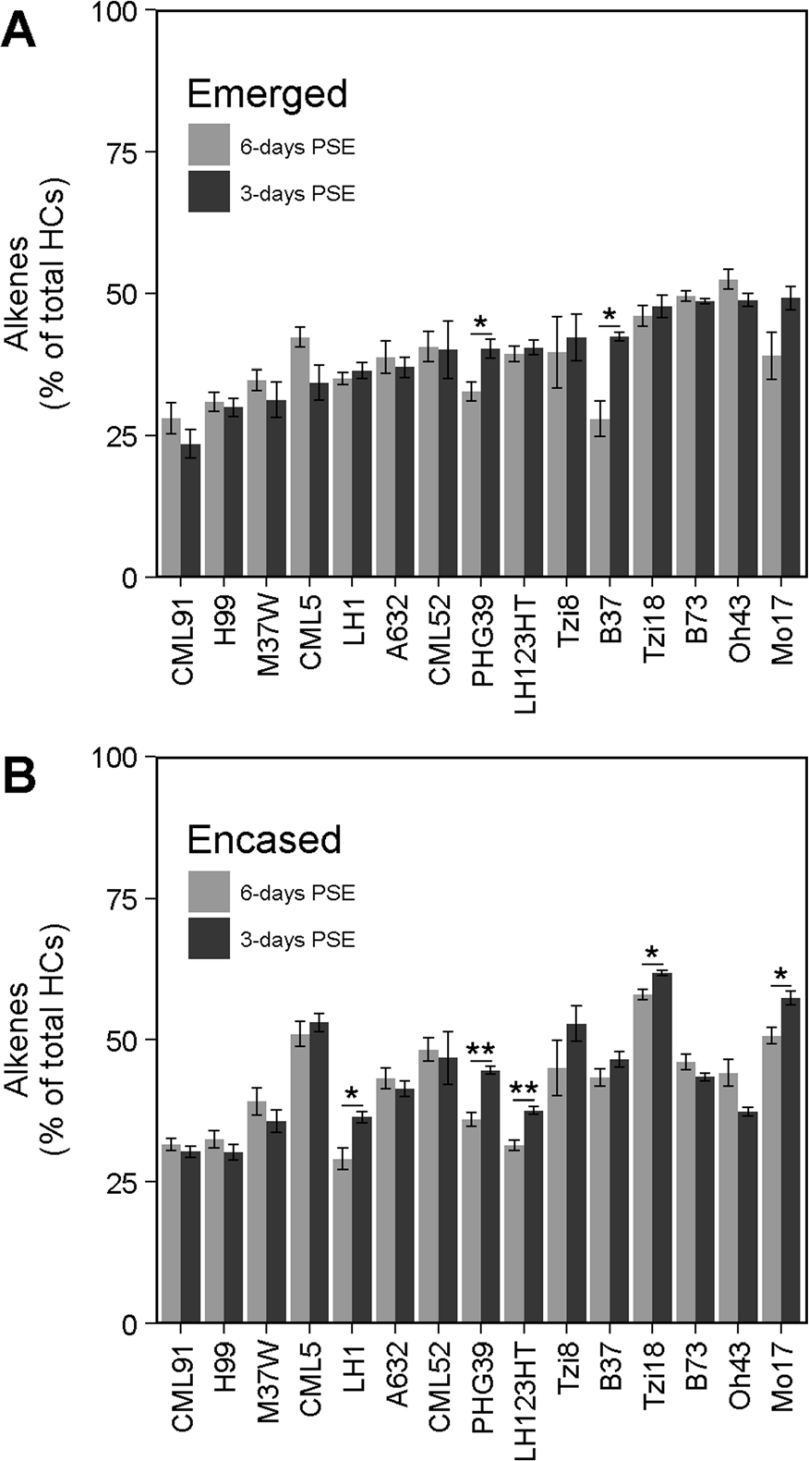
Relative composition of alkenes on silks collected at 3- and 6-days PSE. **a** and **b** Percentage of alkenes relative to total hydrocarbons for emerged (**a**) and husk-encased (**b**) silk sections from 15 inbred lines grown in 2009. Inbred lines are ordered from low to high percentage alkenes on emerged silks at 3-days PSE. Asterisks indicate a significant difference between the percentage of alkenes at 3- and 6-days PSE on emerged (**a**) or husk-encased (**b**) silks of a given inbred line (T-test; * *P* < 0.05, **P < 0.001). Error bars represent ± standard error. HCs, hydrocarbons

**Fig. 6:**
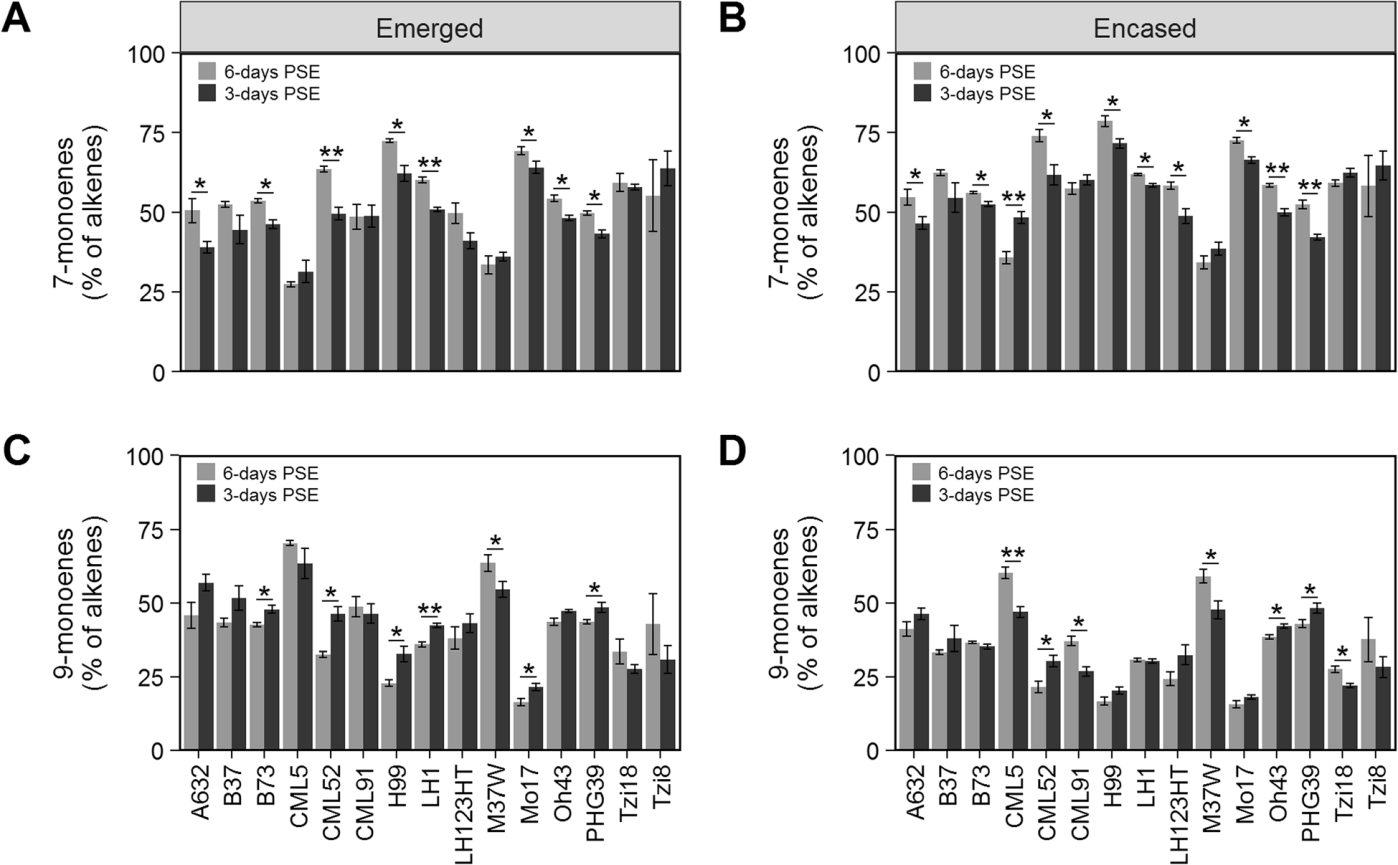
7- and 9-monoene accumulation relative to total alkenes on silks at 3- and 6-days PSE. **a-d** Mean percentage of 7-monoenes on emerged (**a**) and husk-encased (**b**) silks and the mean percentage of 9-monoenes on emerged (**c**) and husk-encased (**d**) silks for 15 inbred lines grown in 2009. Inbred lines are ordered alphabetically in all panels. Asterisks indicate a significant difference between 3- and 6-days PSE for emerged or husk-encased silks of a given inbred line (T-test; * *P* < 0.05, ** *P* < 0.001). Error bars represent ± standard error

Duration of exposure to the external environment was shown to have a minor influence on relative alkene composition (partial *R*^2^ = 0.04; Additional file 4: Table S3). Although alkene composition did not vary for most inbred lines, specific genotypes exhibited lower percentages of alkenes at 6-days PSE (Fig. 5). Similar to the relative abundance of alkenes, the relative compositions of 7- and 9-monoenes, specifically, are dynamically influenced by genotype (partial R^2^ ≈ 0.60), with encasement status and duration of environmental exposure minimally explaining the observed variation (Additional file 8: Table S4 and Additional file 9: Table S5). The interaction of inbred genotype and duration of exposure to the external environment explain minor amounts of the observed variation for composition of 7- and 9-monoenes, as well as for the relative abundance of alkenes more generally (see genotype X PSE interaction term of ANOVA models in Additional file 4: Table S3, Additional file 8: Table S4, and Additional file 9: Table S5). For example, emerged silks from six inbred lines (B73, CML52, H99, LH1, Mo17, and PHG39) exhibited 5 to 15% higher relative abundance of 7-monoenes (Fig. 6a) and a concomitant decrease in the percentage of 9-monoenes at 6-days PSE (Fig. 6c). Similarly, for husk-encased silks from four inbred lines (CML5, CML52, Oh43, and PHG39) we observed that the percentage of 7-monoenes was higher at 6-days PSE (Fig. 6b) with an associated decrease in the percentage of 9-monoenes (Fig. 6d). This might suggest a metabolic shift in the usage and/or availability of specific unsaturated fatty acid precursors in the hydrocarbon biosynthesis pathway that leads to higher accumulation of 7-monoenes as exposure to the external environment increases. Collectively, these data suggest that the fatty acid desaturation pathways may be differentially influenced by increased duration of environmental exposure in some inbred lines.

### Field environment affects the silk surface hydrocarbon metabolome in a genotype-dependent manner

To test the hypothesis that environmental conditions can impact the composition of the metabolome, we surveyed seven inbred lines in two consecutive growing years (i.e. field environments; Table 1). Weather datasets collected near the field sites and reported at Iowa Environmental Mesonet [36] were downloaded for the period from the time of planting to the time of silk collection. Environmental conditions varied substantially between the 2 years. The 2009 and 2010 field locations received 36 cm and 74 cm of rainfall, respectively. In addition, average high and low temperatures were each approximately 3 °C higher in 2010, making 2010 a mildly warmer and significantly wetter year.

**Fig. 7:**
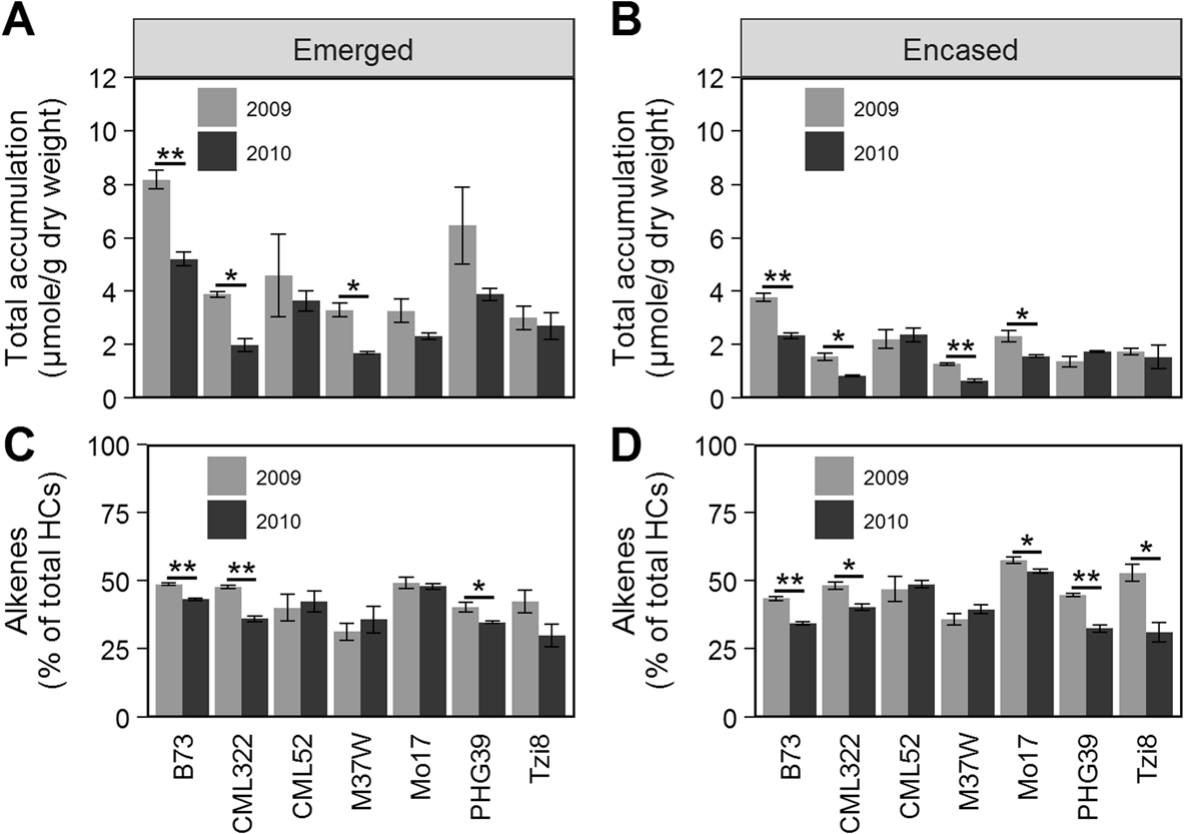
Hydrocarbon accumulation on silks of seven inbred lines grown in two consecutive years. Mean total hydrocarbon accumulation (**a** and **b**) and relative alkene composition (**c** and **d**) for emerged (**a** and **c**) and husk-encased (**b** and **d**) silks collected at 3-days PSE. Inbred lines are ordered alphabetically in all panels. Asterisks indicate a significant difference between growing years (i.e. field environments) for emerged or husk-encased silks of a given inbred line (T-test; * *P* < 0.05, ** *P* < 0.001). Error bars represent ± standard error. HCs, hydrocarbons

Four of the evaluated inbred lines showed a field environment response for total hydrocarbons, with B73, CML322, and M37W exhibiting 1.6- to 2.0-fold higher accumulation for both emerged and husk-encased silks in the 2009 experiment (Fig. 7a and b). Mo17 showed a 1.5-fold increase in hydrocarbon accumulation in 2009 (i.e. cooler and drier growing year), however only for husk-encased silks. Statistical evaluation of these datasets (Additional file 3: Table S2) indicated that genotype and silk encasement status explain the majority of the variation observed in hydrocarbon accumulation (partial *R*^2^ = 0.29 and 0.25, respectively). Although field environment also contributes to the observed variation (partial *R*^2^ = 0.06), it is to a lesser extent. Two-way interactions between genotype and encasement status (partial *R*^2^ = 0.08), and encasement status and field environment (partial *R*^2^ = 0.02) also contribute, but again in relatively small amounts. In addition, a two-way interaction between inbred genotype and field environment (partial *R*^2^ = 0.03) supports statistically-significant albeit minor *GxE* interactions for total accumulation of the surface hydrocarbon metabolome.

Similarly, the relative composition of the surface hydrocarbon metabolome was also influenced by field environment variation. For example, B73, CML322, and PHG39 showed approximately 6 to 12% higher alkene abundance in the drier and cooler 2009 field environment, and this increase was irrespective of whether the silks were husk-encased or emerged (Fig. 7c and d). In contrast, few significant differences were observed in the relative composition of 7- and 9-monoenes between these 2 years. Statistical analysis (Additional file 4: Table S3) indicated that field environment variation and husk leaf encasement status are relatively small contributors (partial *R*^2^ = 0.09 and 0.02, respectively) to compositional changes in the silk surface hydrocarbon metabolome, whereas genotype has the largest effect (partial *R*^2^ = 0.32). Additionally, two-way interactions between inbred genotype and husk leaf encasement status (partial *R*^2^ = 0.08), and genotype and field environment (partial *R*^2^ = 0.11), are small but significant, reinforcing the existence of *GxE* interactions in determining the composition of the surface hydrocarbon metabolome.

## Discussion

### Genotype, silk micro-environment and field environment impact the surface hydrocarbon metabolome

Using a panel of 32 diverse maize inbred lines that belong to five independent heterotic groups, we demonstrate that genetics, silk micro-environment and *GxE* interactions impact both total hydrocarbon accumulation and metabolome composition. PCA reveals that the observed diverse surface lipid compositions do not cluster according to heterotic group. However, we observe that the B73 surface hydrocarbon metabolome is unique, which may have broad importance in commercial corn production. B73 has been the single most important contributor of alleles to commercial germplasm since its public release in 1972 [37], contributing more than 12% of the genomes of the 1132 corn inbred lines registered between 1984 and 2008 [38]. Moreover, B73 germplasm has been particularly important for ear parents in single-cross hybrid seed production, wherein seed number, seed size and seed quality are all important traits to maintain. We consider it possible that B73’s unique silk surface metabolome may in part account for its superiority as a female parent.

Extracellular surface lipid (i.e. cuticular lipid) composition has previously been shown to vary between emerged and husk-encased portions of maize silks [18, 19]. In both growing years of this study, nearly all of the evaluated inbred lines exhibited higher surface hydrocarbon accumulation on emerged versus husk-encased silks. A genotype-by-silk micro-environment interaction was identified, indicating that inbred lines respond differently to the conditions experienced by silks that have emerged into the external environment. Alternatively, silk development may play a role, because cell division and elongation have ceased in emerged silks [39] at 3-days PSE, such that they may be developmentally different than their husk-encased counterparts. Increased hydrocarbon accumulation on emerged silks has previously been observed in studies involving a limited number of inbred lines [18, 19, 21]. Emerged and husk-encased silks from B73 and Mo17 can be compared to a previous study [18]. In the current study, B73 and Mo17 accumulated 2.2- and 1.4-fold more hydrocarbons on emerged silks at 3-days PSE, whereas the previous study observed 3.8- and 2.5-fold higher accumulation on emerged silks, respectively [18]. The differences observed between these studies might be attributable to different environments, which are again indicative of genotype-environment (*GxE*) interactions.

Surface hydrocarbon accumulation varied between two durations of exposure to the external environment, 3- and 6-days PSE. Consistent with our results for 15 diverse inbred lines sampled at these two durations, a previous report has shown that a smaller panel evaluated at increasing days PSE exhibited different patterns of alkane accumulation from 2- to 8-days PSE [21]. Previous studies have shown an increase in total hydrocarbon accumulation for both husk-encased and emerged silks from inbred B73 harvested at 3-days as compared to 2-days PSE and less drastic increases through 7-days PSE [19], and approximately 2-fold more hydrocarbons on emerged and husk-encased silks from inbred lines B73 and Mo17 at 6-days PSE as compared to 3-days PSE [18]. In this study, however, no significant differences in total hydrocarbon accumulation were observed for silks from inbred lines B73 and Mo17 exposed to the external environment for different durations, which is suggestive of environmental (e.g. field environment) effects.

Indeed, our comparison of seven inbred lines evaluated in two growing years revealed differences in surface hydrocarbon metabolome composition. We observed increased total accumulation of hydrocarbons as well as an increase in the relative abundance of alkenes in several of the inbred lines grown during the drier and cooler 2009 season. Interestingly, water deficit has been shown to increase cuticular hydrocarbon accumulation on leaves in Arabidopsis [26], soybean [23], and sesame [24], which is consistent with a protective role for these metabolites against environmental stress. This study expands and strengthens the observation of significant *GxE* effects on the silk surface hydrocarbon metabolome, which were made in a previous report using fewer hydrocarbon metabolites from a smaller and less diverse set of inbred lines [21].

### Even-numbered acyl chain length hydrocarbons suggest alternative precursors for hydrocarbon biosynthesis

Hydrocarbon biosynthesis is widely accepted to initiate from even-numbered acyl chain length VLCFA precursors, and via a decarbonylation reaction they are converted to the corresponding hydrocarbons with one fewer carbon atoms. Although odd-numbered chain length hydrocarbons dominate the metabolomic landscape on silks, even-numbered acyl chain length hydrocarbons were also observed, and abundances varied across inbred lines, particularly for emerged silks. The consistent presence of even-numbered hydrocarbons across the dataset suggests that odd-numbered VLCFAs may also serve as precursors in the decarbonylation-based hydrocarbon biosynthesis pathway. Indeed, prior studies have detected odd-numbered chain length fatty acids (e.g. heptadecanoic acid, tricosanoic acid) on the surfaces of maize silks [18, 20], and corresponding even-numbered hydrocarbons that could result from these precursors (i.e. hexadecane and docosane) [18].

### Alkene composition and potential differential roles of acyl-ACP desaturases

Alkene composition varies widely among inbred lines, comprising 5 to 57% of surface hydrocarbons depending on genotype and husk-encasement status in 2010. Even with this wide breadth of variation, it remains unclear whether alkenes confer biological functions that differ from alkanes. Interestingly, studies that have shown that drought conditions induce accumulation of cuticular hydrocarbons have focused on organisms or tissues that produce appreciable amounts of alkanes, and not alkenes (e.g. Arabidopsis, soybean, sesame) [23, 24, 26]. The prevalent accumulation of alkenes on silks and the rich diversity in alkene compositions may provide a tractable system for assessment of potential differences in function for cuticular alkanes versus alkenes, which would have applications in other plants that accumulate alkenes (e.g. barley) [40].

Within the alkene portion of the metabolome, the abundances of specific alkene classes (i.e. 7- and 9-monoenes) varied based on genotype. Acyl-ACP desaturases, which introduce carbon-carbon double bonds within saturated acyl-ACP substrates, display different specificities, by either acting on substrates of different acyl-chain lengths (e.g. 16- or 18-carbon acyl chains) and/or by removing hydrogen atoms from pairs of adjoining carbon atoms that are differently positioned in a given acyl-chain. For example, the archetypal desaturase in this class of enzymes is the stearoyl-ACP desaturase, which is specific for the saturated 18-carbon fatty acyl-ACP (i.e. stearoyl-ACP), and removes hydrogen atoms from the 9th and 10th carbon atoms, generating oleoyl-ACP (i.e. ∆^9^-octadecenoyl-ACP) (reviewed in [30]). Subsequently, via the endoplasmic reticulum-associated fatty acid elongation pathway, the resulting oleoyl-CoA intermediate can be successively elongated to yield VLCFA intermediates ranging in size from 20 to more than 30 carbons in length. These VLCFA intermediates can be reduced to the corresponding aldehyde and subsequently decarbonylated to form the 9-monoene series of alkenes. Alternatively, a palmitoyl-ACP desaturase has been identified in several plant species including Arabidopsis [41, 42], cat’s claw (*Doxantha unguis-cati* L.) [43], and milkweed (*Asclepias syriaca*) [44], that converts the 16-carbon palmitoyl-ACP to palmitoleoyl-ACP (i.e. ∆^9^-hexadecenoyl-ACP), which would generate the 7-monoene series of alkenes. Indeed, both the 18-carbon stearic acid and corresponding monounsaturated oleic acid (i.e. ∆^9^-octadecenoic acid), as well as the 16-carbon palmitic acid and the corresponding monounsaturated palmitoleic acid (i.e. ∆^9^-hexadecenoic acid) accumulate on maize silks [18, 19]. There are other possible permutations for generating the 7- and 9-monoene series of hydrocarbons. For example, a stearoyl-ACP desaturase could act on the same substrate (i.e. stearoyl-ACP), but remove hydrogen atoms from the 11th and 12th carbon atoms, which by analogy would generate ∆^11^-octadecenoyl-ACP, ultimately giving rise to the 7-monoene series of alkenes. While a stearoyl-ACP desaturase with this substrate specificity has not been identified, the monounsaturated vaccenic acid (i.e. ∆^11^-octadecenoic acid) that would result from this proposed pathway has been observed on maize silks [18, 19]. Examination of 11 maize *sacd* genes [31] revealed that six of these harbored amino acid polymorphisms between B73 and Mo17 that may influence the formation of the functional homodimer or otherwise impact functionality. Importantly, these polymorphisms may underlie the variations in alkene composition observed in this study and provide candidate *sacd* genes to functionally characterize.

## Conclusions

This study presents a detailed dissection of the surface hydrocarbon landscape on silks from 32 genetically diverse maize inbred lines, identifying wide variation in total hydrocarbon accumulation and the relative composition of the metabolome. Within many of the inbred lines, metabolome composition varies between emerged and husk-encased portions of silks, which are exposed to very different micro-environments, demonstrating a likely impact of silk environment on the genetic pathways responsible for the surface hydrocarbon metabolome. The observed increase in hydrocarbon accumulation on emerged silks for most inbred lines is consistent with a potential protective role for hydrocarbons against environmental stresses. Indeed, different environmental conditions between growing years altered the accumulation of hydrocarbons on the silk surface and inbred lines responded to these stimuli differently. It remains an open question of what specific environmental stimuli are responsible for the observed effects on maize silks, at what stage of maize development these stimuli are influential and the extent to which silk development plays a role in influencing the surface hydrocarbon metabolome. Even so, this work lays a foundation for future studies that pursue the functional significance of alkane and alkene compositions and whether specific compositions protect against certain biotic or abiotic stresses (e.g. insect herbivory, drought).

The metabolome variation observed across this diverse set of germplasm is largely explained by genotype and includes an 11-fold difference in total hydrocarbon accumulation and appreciable variation in other hydrocarbon composition traits, providing a foundation for genetic mapping strategies to identify controlling genetic loci underlying cuticular surface lipid biosynthesis. Two existing synergistic approaches in maize that utilize diverse inbreds lines shown herein to have unique hydrocarbon metabolomes include nested association mapping using populations derived from the NAM founder lines [27] and biparental linkage mapping with the intermated B73xMo17 mapping population [45]. Ultimately, the coordinated understanding of the controlling loci and how the cuticular metabolome protects against environmental stresses might facilitate future applied breeding for specific metabolome compositions that exhibit enhanced protection from specific environmental stresses.

## Methods

### Plant material and tissue sampling

A subset of 19 maize (*Zea mays* L.) founder lines of the NAM population [27] were obtained from the North Central Regional Plant Introduction Station (Ames, IA). Seeds from inbred lines PHG39, PHG84, LH1 and LH123T were kindly donated by Bill Beavis (Iowa State University) and Martin Bohn (Illinois University). During the summer of 2009, 18 inbred lines were cultivated at the Iowa State University Agronomy Farm (Boone, IA) and 22 inbred lines were grown at the Iowa State University Curtiss Farm (Ames, IA) in 2010 (Table 1). In both years of the study, inbred lines were planted in a completely randomized design using common cultivation practices and no supplemental irrigation. B73, CML322, CML52, M37W, Mo17, PHG39, and Tzi8 were examined in both years of the study.

Ear shoots were covered prior to silking to prevent pollination, and silk emergence from the husk leaves was monitored daily. For sample collection, immature ears were harvested between 10 am and 12 pm either 3- or 6-days PSE and were transported to the laboratory on ice. For the 2009 study, a minimum of three biological replicates were harvested for each inbred line, with the majority of lines having five or six biological replicates. For the 2010 study, a minimum of three biological replicates were harvested per line, with the majority having five or more biological replicates. The agronomically important inbred lines, B73 and Mo17 were more deeply replicated, with more than ten biological replicates each (Table 1). Emerged silks from each biological replicate were excised at the point of emergence from the encasing husk leaves. After husking the ear, the husk-encased silks were carefully removed from the cob to ensure that ovule tissue was not collected. Silk samples were flash-frozen in liquid nitrogen and stored at − 80 **°**C. The samples were subsequently lyophilized in a FreeZone 4.5 Liter Freeze Dry System (Labconco, MO) and pulverized with a Geno/Grinder 2000 (Spex CertiPrep, NJ). The mass of the silk powder was recorded for metabolite accumulation calculations.

### Extraction of hydrocarbons

Immediately prior to hydrocarbon extraction, 10 μg of hexacosane (1 mg/mL) (Fluka, WI) was applied directly to the dried plant material (ca. 100 mg of tissue) and served as an internal standard. Hydrocarbons from each sample were extracted in 1.5 mL HPLC-grade hexanes (Fisher Scientific, NJ) with sonication for 15 min and centrifuged at 13,000 *X g* for 1 min. Supernatant was collected after each centrifugation, and the extraction, sonication, and centrifugation cycle was repeated three times. The combined supernatant was passed through a glass pipette containing 0.6 g silica gel (J.T. Baker, NJ) that was pre-washed with 10 mL hexanes. After elution, the silica gel was washed with 10 mL hexanes. The combined eluents were evaporated under a stream of N_2_ gas in a rotary nitrogen evaporator (Organomation Associates, Inc., MA) at 30 °C. It has been previously shown that there is no significant difference in hydrocarbon content between silk samples extracted after being dried and powdered, and fresh silk samples dipped in the extraction solvent [19]. Therefore, we conclude that the extracted hydrocarbons resided on the silk surface. The dried hydrocarbon samples were dissolved in 1 mL hexanes prior to analysis via GC-MS.

### Derivatization of unsaturated hydrocarbons

Double-bond positions of identified alkenes were determined by mass spectrometric analysis of dimethyl disulfide adducts as reported [46], with modifications. Extracts were incubated in 50 μl of hexanes, 200 μl of dimethyl disulfide, and 50 μl of an iodine solution (6% w/v in diethyl ether) for 24 h at 40 °C. The reaction was terminated by addition of 500 μl of sodium thiosulfate (5% w/v), and the derivatized lipids were phase-extracted in 500 μl of hexanes. Extracts were concentrated under a stream of N_2_ gas and analyzed via GC-MS.

### Gas chromatography-mass spectrometry

Chromatographic analysis of samples collected in 2009 and 2010 was performed in year 2 of the study at the W.M. Keck Metabolomics Research Laboratory at Iowa State University. A gas chromatograph (GC; Model 6890 series, Agilent Technologies, Palo Alto, CA), equipped with a mass detector (Model 5973, Agilent Technologies, Palo Alto, CA) was used. Chromatography was conducted with a HP-5MS cross-linked (5%)-diphenyl-(95%)-dimethyl polysiloxane column (30 m length, 0.25 mm inner diameter) using helium as the carrier gas. For underivatized hydrocarbon extracts, 1 μl aliquots were injected into the GC via splitless injection and the GC oven temperature program initiated at 200 **°**C, was increased to 280 **°**C at a rate of 4 **°**C/min, further increased to 320 **°**C at a rate of 20 **°**C/min and held at this temperature for 3 min. For extracts derivatized with dimethyl disulfide, 1 μl aliquots were injected into the GC via splitless injection and the GC oven temperature program started at 120 **°**C, was increased to 160 **°**C at a rate of 10 **°**C/min and held at this temperature for 2 min, further increased to 260 **°**C at a rate of 5 **°**C/min and held at this temperature for 10 min, further increased to 300 **°**C at a rate of 5 **°**C/min and held at this temperature for 5 min, further increased to 320 **°**C at a rate of 5 **°**C/min and was held at this temperature for 17 min.

To quantify hydrocarbon constituents, the response of the mass-spectrometer was calibrated to the hexacosane internal standard within each sample. Quantification analysis was performed using the AMDIS software package [47] and compounds were identified via the National Institute of Standards and Technology Mass Spectral Library [48]. All hydrocarbon accumulation data are provided in the supplementary materials (Additional file 1: Table S1).

The limit of detection for GC-MS analysis was 0.0034 μmole/g dry weight. Accumulation values greater than zero and less than the limit of detection were modified to 0.0017 for analyses. To determine the limit of detection, the accumulation of the hydrocarbon with the lowest mean for each genotype, growing year (i.e. field environment), and days PSE combination that had a relative standard deviation of less than 33% and a mean of less than 0.005 μmole/g dry weight was identified and the limit of detection was calculated as the average of these identified mean values.

### Statistical methods

All metabolite data were gathered from a minimum of three biological replicates (Table 1). Student’s t-tests assuming unequal variance were conducted using version 3.4.3 of R [49] for pairwise comparisons of mean metabolite accumulation for one inbred line between emerged and husk-encased silks, between 3- and 6-days PSE time points for either emerged or husk-encased silks, and between growing years for either emerged or husk-encased silks. ANOVA (e.g. two-way and three-way) was conducted on appropriately partitioned data sets derived from Additional file 1: Table S1 to evaluate the effects of genotypic and environmental factors (Additional file 3: Table S2, Additional file 4: Table S3, Additional file 8: Table S4, Additional file 9: Table S5, Additional file 12: Table S7) using JMP Pro (version 12.0.1, SAS Institute Inc., Cary, NC). PCA was conducted to explore silk surface lipid accumulation patterns across heterotic groups using the prcomp() function in the R/stats base package [49], and 95% confidence ellipses were constructed using the stat_ellipse() function in the R/ggplot2 package (Fig. 2) [50].

## Supplementary information

**Additional file 1: Table S1.** Abundances of all hydrocarbon constituents for silk samples from inbred lines grown in 2009 and 2010. Individual metabolite abundances (μmol/g dry weight) are provided for each silk extract in this study, which includes emerged and husk-encased silk extracts from 32 inbred lines evaluated at either or both 3-days and 6-days post-silk emergence in either or both the 2009 and 2010 growing seasons. A key to column headings is provided in the file.

**Additional file 2: Figure S1.** Total hydrocarbon accumulation on silks collected 3-days PSE from inbred lines grown in 2009. Inbred lines are ordered from lowest to highest hydrocarbon accumulation on emerged silks. Asterisks indicate hydrocarbon accumulation on emerged silks is significantly greater than husk-encased silks for a given inbred line (T-test; * *P* < 0.05), ** *P* < 0.001). Error bars represent ± standard error.

**Additional file 3: Table S2.** ANOVAs of total hydrocarbon accumulation. Two-way ANOVA assessed the effects of genotype and husk-encasement status at 3-days PSE in both growing years and at 6-days PSE in 2009. A three-way ANOVA assessed the effects of genotype, husk-encasement status and days PSE for growing year 2009 and a second three-way ANOVA assessed the effects of genotype, husk-encasement status and growing year for silk samples harvested at 3-days PSE in both growing years.

**Additional file 4: Table S3.** ANOVAs of the percentage of alkenes relative to total hydrocarbon accumulation. Two-way ANOVA assessed the effects of genotype and husk-encasement status at 3-days PSE in both growing years and at 6-days PSE in 2009. A three-way ANOVA assessed the effects of genotype, husk-encasement status and days PSE for growing year 2009 and a second three-way ANOVA assessed the effects of genotype, husk-encasement status and growing year for silk samples harvested at 3-days PSE in both growing years.

**Additional file 5: Figure S2.** Percentage of alkenes on silks collected 3-days PSE from inbred lines grown in 2009. Inbred lines are ordered from low to high mean percentage of alkenes relative to total hydrocarbon accumulation on emerged silks. Asterisks indicate a significant difference between emerged and husk-encased silks of a given inbred line (T-test; * *P* < 0.05, ** *P* < 0.001). Error bars represent ± standard error. HCs, hydrocarbons.

**Additional file 6: Figure S3.** Mass-spectral identification of silk surface alkenes. The double bond positions of monoenes were determined from GC-MS analysis of dimethyl disulfide adducts of unsaturated metabolites in the hydrocarbon extracts. **A.** The identification of 7-nonacosene is shown. The fragmentation of 7-nonacosene generates daughter ions of 145 [(C_8_H_17_S)+] and 355 [(C_23_H_47_S)+] *m/z* units, identifying the double bond position to be between the 7th and 8th carbon atoms of the alkyl chain. **B.** The identification of 9-nonacosene is shown. The fragmentation of 9-nonacosene generates daughter ions of 173 [(C_8_H_17_S)+] and 327 [(C_23_H_47_S)+] *m/z* units, identifying the double bond position between the 9th and 10th carbon atoms of the alkyl chain.

**Additional file 7: Figure S4.** Variation in 7- and 9-monoene accumulation relative to total alkenes on silks. Mean percentage of 7-monoenes (**A**) and 9-monoenes (**B**) on silks from 22 inbred lines grown in 2010 and analyzed at 3-days PSE. Inbred lines are ordered by increasing percentage of 7-or 9-monoenes on emerged silks in panels A and B, respectively. Asterisks indicate a significant difference between emerged and husk-encased silk means for a given inbred line (T-test; * *P* < 0.05, ** *P* < 0.001). Error bars represent ± standard error.

**Additional file 8: Table S4.** ANOVAs of the percentage of 7-monoenes relative to total alkenes. Two-way ANOVA assessed the effects of genotype and husk-encasement status at 3-days PSE in both growing years and at 6-days PSE in 2009. A three-way ANOVA assessed the effects of genotype, husk-encasement status and days PSE for growing year 2009 and a second three-way ANOVA assessed the effects of genotype, husk-encasement status and growing year for silk samples harvested at 3-days PSE in both growing years.

**Additional file 9: Table S5.** ANOVAs of the percentage of 9-monoenes relative to total alkenes. Two-way ANOVA assessed the effects of genotype and husk-encasement status at 3-days PSE in both growing years and at 6-days PSE in 2009. A three-way ANOVA assessed the effects of genotype, husk-encasement status and days PSE for growing year 2009 and a second three-way ANOVA assessed the effects of genotype, husk-encasement status and growing year for silk samples harvested at 3-days PSE in both growing years.

**Additional file 10: Table S6.** Polymorphic amino acids between B73 and Mo17 alleles of *sacd* genes. *Sacd* syntelogs in the Mo17 and B73 genomes were obtained from precomputed resources at MaizeGDB [51, 52] and amino acid polymorphisms were identified via sequence alignments. Specific single amino acid and insertion/deletion polymorphisms between the two alleles are presented.

**Additional file 11: Figure S5.** Percentage of even-numbered chain lengths relative to total hydrocarbon accumulation. Percentage of hydrocarbons having even-numbered acyl chain lengths on silks from inbred lines grown in 2010 **(A**) and 2009 **(B**) and analyzed at 3-days PSE**.** Inbred lines are ordered from low to high percentage for emerged silks. Asterisks indicate a significant difference between emerged and husk-encased silk means of a given inbred line (T-test; * *P* < 0.05, ** *P* < 0.001). Error bars represent ± standard error. IL14H, grown in 2010, did not accumulate observable amounts of even-numbered chain length hydrocarbons (**A**). HCs, hydrocarbons.

**Additional file 12: Table S7.** ANOVAs of the percentage of even-numbered chain length hydrocarbons relative to total hydrocarbon accumulation. Two-way ANOVA assessed the effects of genotype and husk-encasement status at 3-days PSE in both growing years and at 6-days PSE in 2009. A three-way ANOVA assessed the effects of genotype, husk-encasement status and days PSE for growing year 2009 and a second three-way ANOVA assessed the effects of genotype, husk-encasement status and growing year for silk samples harvested at 3-days PSE in both growing years.

## Data Availability

All data generated or analyzed during this study are included in this published article and its supplementary information files.

## Abbreviations

ACP: Acyl carrier protein
ANOVA: Analysis of variance
ex-PVP: Ex-plant variety protection
GC-MS: Gas chromatography-mass spectrometry
GWAS: Genome-wide association study
GxE: Genotype x Environment interaction
NAM: Nested association mapping
ORF: Open reading frame
PCA: Principal component analysis
PSE: Post-silk emergence
QTL: Quantitative trait locus
SACD: Stearoyl-ACP desaturase
VLCFA: Very long chain fatty acid
