# Supplementary material for: Genetic and environmental variation impact the cuticular hydrocarbon metabolome on the stigmatic surfaces of maize
Source: BMC Plant Biol. 2019 Oct 17;19:430. doi: 10.1186/s12870-019-2040-3 (PMC6796380; doi:10.1186/s12870-019-2040-3)
Supplement: Supplementary file 2 — Additional file 2: Figure S1. Total hydrocarbon accumulation on silks collected 3-days PSE from inbred lines grown in 2009. Inbred lines are ordered from lowest to highest hydrocarbon accumulation on emerged silks. Asterisks indicate hydrocarbon accumulation on emerged silks is significantly greater than husk-encased silks for a given inbred line (T-test; * P < 0.05), ** P < 0.001). Error bars represent ± standard error. [file 12870_2019_2040_MOESM2_ESM.pdf]

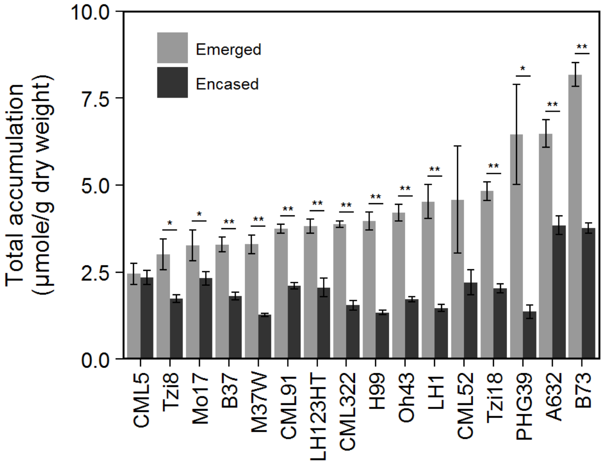

Figure S1. Total hydrocarbon accumulation on silks collected 3-days PSE from inbred lines grown in 2009. Inbred lines are ordered from lowest to highest hydrocarbon accumulation on emerged silks. Asterisks indicate hydrocarbon accumulation on emerged silks is significantly greater than husk-encased silks for a given inbred line (T-test; * $P<0.05$), ** $P<0.001$). Error bars represent ± standard error.
