# Supplementary material for: Genetic and environmental variation impact the cuticular hydrocarbon metabolome on the stigmatic surfaces of maize
Source: BMC Plant Biol. 2019 Oct 17;19:430. doi: 10.1186/s12870-019-2040-3 (PMC6796380; doi:10.1186/s12870-019-2040-3)
Supplement: Supplementary file 3 — Additional file 3: Table S2. ANOVAs of total hydrocarbon accumulation. Two-way ANOVA assessed the effects of genotype and husk-encasement status at 3-days PSE in both growing years and at 6-days PSE in 2009. A three-way ANOVA assessed the effects of genotype, husk-encasement status and days PSE for growing year 2009 and a second three-way ANOVA assessed the effects of genotype, husk-encasement status and growing year for silk samples harvested at 3-days PSE in both growing years. [file 12870_2019_2040_MOESM3_ESM.pdf]

Table S2. ANOVAs of total hydrocarbon accumulation.

<table>
<tr><td>Year[a]</td><td>Days PSE[b]</td><td>Two-way ANOVA[c]</td><td>Three-way ANOVA with days PSE effect[d]</td><td>Three-way ANOVA with growing year (field environment) effect[e]</td></tr>
<tr><td rowspan="2">2009</td><td>6</td><td>**Genotype:**
$F_{16,142}$=15.64, P<0.0001, partial R^2=0.26

Encasement status:
$F_{1,142}$=483.32, P<0.0001, partial R^2=0.49

Genotype X Encasement status:
$F_{16,142}$=5.06, P<0.0001, partial R^2=0.08

R^2=0.86</td><td rowspan="2">**Genotype:**
$F_{14,325}$=22.66, P<0.0001, partial R^2=0.25

Encasement status:
$F_{1,325}$=538.84, P<0.0001, partial R^2=0.49

Days PSE[b]:
$F_{1,325}$=6.63, P=0.0106, partial R^2=0.01

Genotype X Encasement status:
$F_{14,325}$=6.64, P<0.0001, partial R^2=0.07

Genotype X Days PSE:
$F_{14,325}$=1.33, P=0.1874, partial R^2=0.01

Encasement status X Days PSE:
$F_{1,325}$=0.97, P=0.3261, partial R^2=0.00

Genotype X Encasement status X Days PSE:
$F_{14,325}$=1.77, P=0.0426, partial R^2=0.02

R^2=0.79</td><td>Not applicable</td></tr>
<tr><td>3</td><td>**Genotype:**
$F_{15,148}$=10.68, P<0.0001, partial R^2=0.28

Encasement status:
$F_{1,148}$=217.65, P<0.0001, partial R^2=0.37

Genotype X Encasement status:
$F_{15,148}$=3.82, P<0.0001, partial R^2=0.10

R^2=0.75</td><td rowspan="2">**Genotype:**
$F_{6,148}$=27.91, P<0.0001, partial R^2=0.29

Encasement status:
$F_{1,148}$=145.61, P<0.0001, partial R^2=0.25

Growing year:
$F_{1,148}$=36.54, P<0.0001, partial R^2=0.06

Genotype X Encasement status:
$F_{6,148}$=7.71, P<0.0001, partial R^2=0.08

Genotype X Growing year:
$F_{6,148}$=2.56, P=0.0217, partial R^2=0.03

Encasement status X Growing year:
$F_{1,148}$=11.32, P=0.0010, partial R^2=0.02

Genotype X Encasement status X Growing year:
$F_{6,148}$=1.21, P=0.3031, partial R^2=0.01

R^2=0.74</td></tr>
<tr><td>2010</td><td>3</td><td>**Genotype:**
$F_{21,198}$ =26.78, P<0.0001, partial R^2=0.46

Encasement status:
$F_{1,198}$=280.74, P<0.0001, partial R^2=0.23

Genotype X Encasement status:
$F_{21,198}$=5.90, P<0.0001, partial R^2=0.10

R^2=0.84</td><td>Not applicable</td></tr>
</table>

[a]Year the inbred lines were grown; [b]Days post-silk emergence (PSE) that the silks were harvested. 3- *versus* 6-days PSE represents two different durations of exposure to the external environment; [c]Two-way full factorial analysis of variance (ANOVA) of the main effects, genotype and encasement status, and their interaction; [d]Three-way full factorial ANOVA of the main effects, genotype, encasement status, and days PSE, and all two- and three-way interactions; [e]Three-way full factorial ANOVA of the main effects, genotype, encasement status, and growing year (*i.e.* field environment), and all two- and three-way interactions; [cde]Main effects are in bold and interaction terms are in italics, F statistics and corresponding p-values are given following the main effects and interaction terms, R^2 values indicate the proportion of variance in total hydrocarbon accumulation explained by the full model and partial R^2 values indicate the proportion of variance explained by each factor.
