# Supplementary material for: Genetic and environmental variation impact the cuticular hydrocarbon metabolome on the stigmatic surfaces of maize
Source: BMC Plant Biol. 2019 Oct 17;19:430. doi: 10.1186/s12870-019-2040-3 (PMC6796380; doi:10.1186/s12870-019-2040-3)
Supplement: Supplementary file 4 — Additional file 4: Table S3. ANOVAs of the percentage of alkenes relative to total hydrocarbon accumulation. Two-way ANOVA assessed the effects of genotype and husk-encasement status at 3-days PSE in both growing years and at 6-days PSE in 2009. A three-way ANOVA assessed the effects of genotype, husk-encasement status and days PSE for growing year 2009 and a second three-way ANOVA assessed the effects of genotype, husk-encasement status and growing year for silk samples harvested at 3-days PSE in both growing years. [file 12870_2019_2040_MOESM4_ESM.pdf]

Table S3. ANOVAs of the percentage of alkenes relative to total hydrocarbon accumulation.

Year[a]	Days PSE[b]	Two-way ANOVA[c]	Three-way ANOVA with days PSE effect[d]	Three-way ANOVA with growing year (field environment) effect[e]
2009	6	**Genotype:**<br>$F_{16,142}=16.95$, $P<0.0001$, partial $R^2=0.53$<br><br>**Encasement status:**<br>$F_{1,142}=25.79$, $P<0.0001$, partial $R^2=0.05$<br><br>*Genotype X Encasement status:*<br>$F_{16,142}=4.69$, $P<0.0001$, partial $R^2=0.15$<br><br>$R^2=0.72$	**Genotype:**<br>$F_{14,266}=38.58$, $P<0.0001$, partial $R^2=0.49$<br><br>**Encasement status:**<br>$F_{1,266}=44.46$, $P<0.0001$, partial $R^2=0.04$<br><br>**Days PSE[b]:**<br>$F_{1,266}=5.42$, $P=0.0207$, partial $R^2=0.01$<br><br>*Genotype X Encasement status:*<br>$F_{14,266}=9.41$, $P<0.0001$, partial $R^2=0.12$<br><br>*Genotype X Days PSE:*<br>$F_{14,266}=4.38$, $P<0.0001$, partial $R^2=0.06$<br><br>*Encasement status X Days PSE:*<br>$F_{1,266}=0.3917$, $P=0.5319$, partial $R^2=0.00$<br><br>*Genotype X Encasement status X Days PSE:*<br>$F_{14,266}=1.24$, $P=0.2494$, partial $R^2=0.02$<br><br>$R^2=0.76$	Not applicable
	3	**Genotype:**<br>$F_{15,148}=25.21$, $P<0.0001$, partial $R^2=0.59$<br><br>**Encasement status:**<br>$F_{1,148}=27.30$, $P<0.0001$, partial $R^2=0.04$<br><br>*Genotype X Encasement status:*<br>$F_{15,148}=6.02$, $P<0.0001$, partial $R^2=0.14$<br><br>$R^2=0.77$		**Genotype:**<br>$F_{6,148}=25.82$, $P<0.0001$, partial $R^2=0.32$<br><br>**Encasement status:**<br>$F_{1,148}=11.13$, $P=0.0011$, partial $R^2=0.02$<br><br>**Growing year:**<br>$F_{1,148}=44.22$, $P<0.0001$, partial $R^2=0.09$<br><br>*Genotype X Encasement status:*<br>$F_{6,148}=6.43$, $P<0.0001$, partial $R^2=0.08$<br><br>*Genotype X Growing year:*<br>$F_{6,148}=9.28$, $P<0.0001$, partial $R^2=0.11$<br><br>*Encasement status X Growing year:*<br>$F_{1,148}=2.80$, $P=0.0966$, partial $R^2=0.01$<br><br>*Genotype X Encasement status X Growing year:*<br>$F_{6,148}=0.85$, $P=0.5358$, partial $R^2=0.01$<br><br>$R^2=0.68$
2010	3	**Genotype:**<br>$F_{21,198}=23.18$, $P<0.0001$, partial $R^2=0.64$<br><br>**Encasement status:**<br>$F_{1,198}=24.32$, $P<0.0001$, partial $R^2=0.03$<br><br>*Genotype X Encasement status:*<br>$F_{21,198}=2.85$, $P<0.0001$, partial $R^2=0.08$<br><br>$R^2=0.74$	Not applicable	

[a]Year the inbred lines were grown; [b]Days post-silk emergence (PSE) when the silks were harvested. 3- *versus* 6-days PSE represents two different durations of exposure to the external environment; [c]Two-way full factorial analysis of variance (ANOVA) of the main effects, genotype and encasement status, and their interaction; [d]Three-way full factorial ANOVA of the main effects, genotype, encasement status, and days PSE, and all two- and three-way interactions; [e]Three-way full factorial ANOVA of the main effects, genotype, encasement status, and growing year (*i.e.* field environment), and all two- and three-way interactions; [cde]Main effects are in bold and interaction terms are in italics, F statistics and corresponding p-values are given following the main effects and interaction terms, R^2 values indicate the proportion of variance in the alkene percentage of total hydrocarbon accumulation explained by the full model and partial R^2 values indicate the proportion of variance explained by each factor.
