# Supplementary material for: Genetic and environmental variation impact the cuticular hydrocarbon metabolome on the stigmatic surfaces of maize
Source: BMC Plant Biol. 2019 Oct 17;19:430. doi: 10.1186/s12870-019-2040-3 (PMC6796380; doi:10.1186/s12870-019-2040-3)
Supplement: Supplementary file 5 — Additional file 5: Figure S2. Percentage of alkenes on silks collected 3-days PSE from inbred lines grown in 2009. Inbred lines are ordered from low to high mean percentage of alkenes relative to total hydrocarbon accumulation on emerged silks. Asterisks indicate a significant difference between emerged and husk-encased silks of a given inbred line (T-test; * P < 0.05, ** P < 0.001). Error bars represent ± standard error. HCs, hydrocarbons. [file 12870_2019_2040_MOESM5_ESM.pdf]

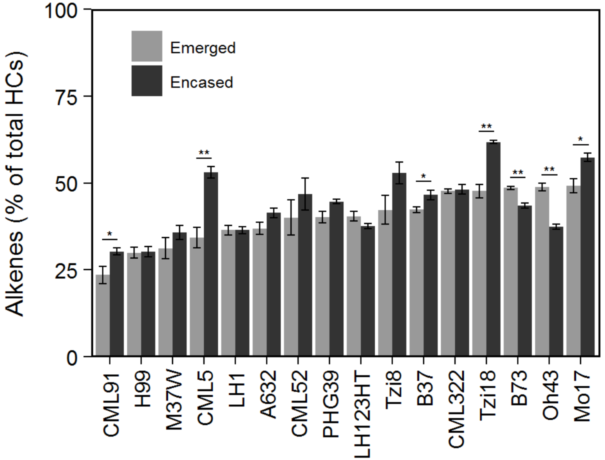

Figure S2. Percentage of alkenes on silks collected 3-days PSE from inbred lines grown in 2009. Inbred lines are ordered from low to high mean percentage of alkenes relative to total hydrocarbon accumulation on emerged silks. Asterisks indicate a significant difference between emerged and husk-encased silks of a given inbred line (T-test; * $P<0.05$, ** $P<0.001$). Error bars represent ± standard error. HCs, hydrocarbons.
