# Supplementary material for: Genetic and environmental variation impact the cuticular hydrocarbon metabolome on the stigmatic surfaces of maize
Source: BMC Plant Biol. 2019 Oct 17;19:430. doi: 10.1186/s12870-019-2040-3 (PMC6796380; doi:10.1186/s12870-019-2040-3)
Supplement: Supplementary file 6 — Additional file 6: Figure S3. Mass-spectral identification of silk surface alkenes. The double bond positions of monoenes were determined from GC-MS analysis of dimethyl disulfide adducts of unsaturated metabolites in the hydrocarbon extracts. A. The identification of 7-nonacosene is shown. The fragmentation of 7-nonacosene generates daughter ions of 145 [(C8H17S)+] and 355 [(C23H47S)+] m/z units, identifying the double bond position to be between the 7th and 8th carbon atoms of the alkyl chain. B. The identification of 9-nonacosene is shown. The fragmentation of 9-nonacosene generates daughter ions of 173 [(C8H17S)+] and 327 [(C23H47S)+] m/z units, identifying the double bond position between the 9th and 10th carbon atoms of the alkyl chain. [file 12870_2019_2040_MOESM6_ESM.pdf]

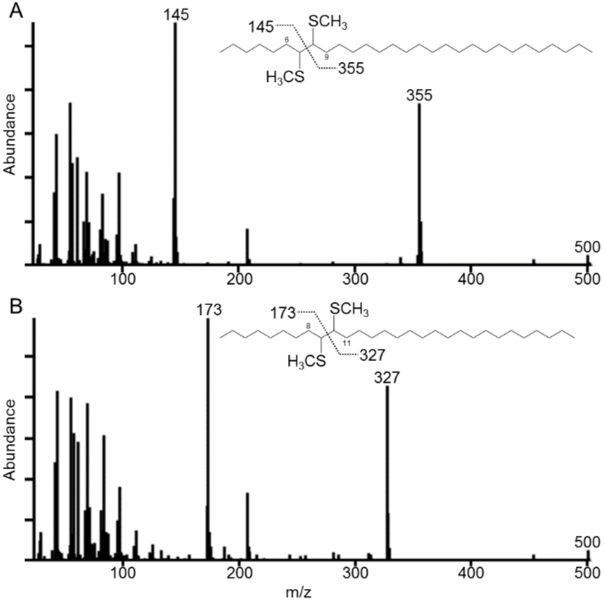

Figure S3. Mass-spectral identification of silk surface alkenes. The double bond positions of monoenes were determined from GC-MS analysis of dimethyl disulfide adducts of unsaturated metabolites in the hydrocarbon extracts. **A.** The identification of 7-nonacosene is shown. The fragmentation of 7-nonacosene generates daughter ions of 145 [$(C_8H_{17}S)+$] and 355 [$(C_{23}H_{47}S)+$] *m/z* units, identifying the double bond position to be between the 7th and 8th carbon atoms of the alkyl chain. **B.** The identification of 9-nonacosene is shown. The fragmentation of 9-nonacosene generates daughter ions of 173 [$(C_8H_{17}S)+$] and 327 [$(C_{23}H_{47}S)+$] *m/z* units, identifying the double bond position between the 9th and 10th carbon atoms of the alkyl chain.
