# Supplementary material for: Genetic and environmental variation impact the cuticular hydrocarbon metabolome on the stigmatic surfaces of maize
Source: BMC Plant Biol. 2019 Oct 17;19:430. doi: 10.1186/s12870-019-2040-3 (PMC6796380; doi:10.1186/s12870-019-2040-3)
Supplement: Supplementary file 7 — Additional file 7: Figure S4. Variation in 7- and 9-monoene accumulation relative to total alkenes on silks. Mean percentage of 7-monoenes (A) and 9-monoenes (B) on silks from 22 inbred lines grown in 2010 and analyzed at 3-days PSE. Inbred lines are ordered by increasing percentage of 7-or 9-monoenes on emerged silks in panels A and B, respectively. Asterisks indicate a significant difference between emerged and husk-encased silk means for a given inbred line (T-test; * P < 0.05, ** P < 0.001). Error bars represent ± standard error. [file 12870_2019_2040_MOESM7_ESM.pdf]

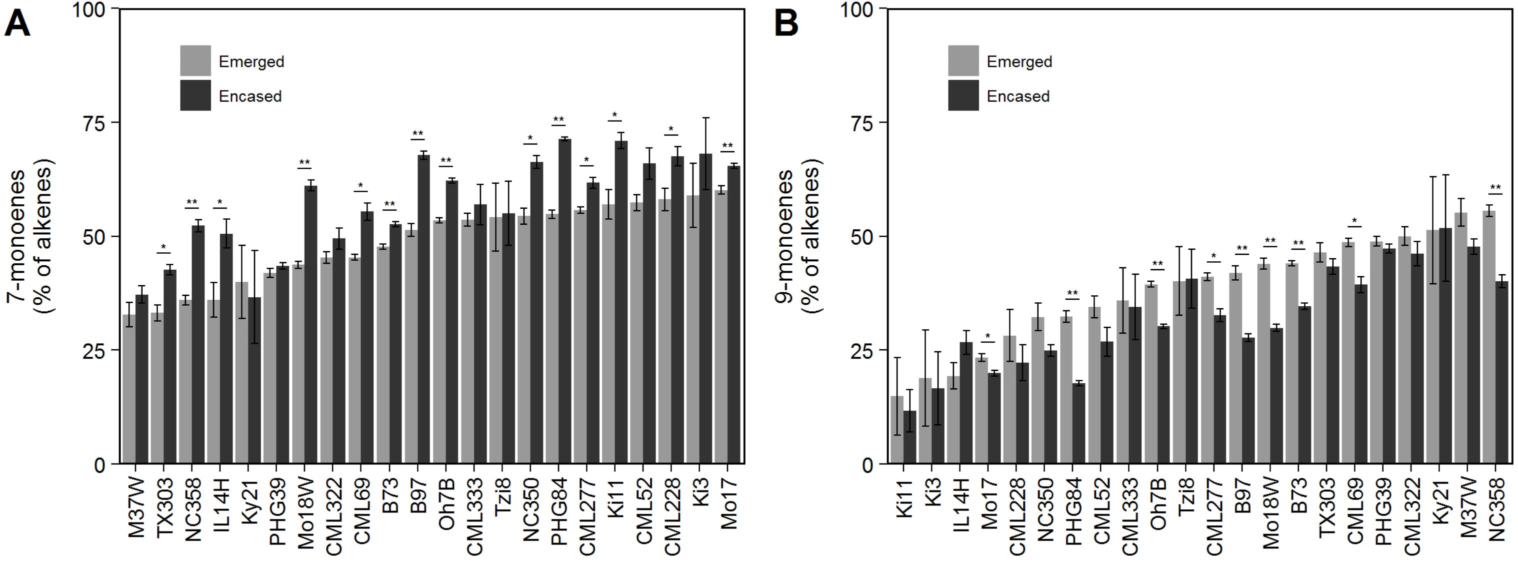

Figure S4. Variation in 7- and 9-monoene accumulation relative to total alkenes on silks. Mean percentage of 7-monoenes (**A**) and 9-monoenes (**B**) on silks from 22 inbred lines grown in 2010 and analyzed at 3-days PSE. Inbred lines are ordered by increasing percentage of 7-or 9-monoenes on emerged silks in panels A and B, respectively. Asterisks indicate a significant difference between emerged and husk-encased silk means for a given inbred line (T-test; * P<0.05, ** P<0.001). Error bars represent ± standard error.
