# Supplementary material for: Genetic and environmental variation impact the cuticular hydrocarbon metabolome on the stigmatic surfaces of maize
Source: BMC Plant Biol. 2019 Oct 17;19:430. doi: 10.1186/s12870-019-2040-3 (PMC6796380; doi:10.1186/s12870-019-2040-3)
Supplement: Supplementary file 9 — Additional file 9: Table S5. ANOVAs of the percentage of 9-monoenes relative to total alkenes. Two-way ANOVA assessed the effects of genotype and husk-encasement status at 3-days PSE in both growing years and at 6-days PSE in 2009. A three-way ANOVA assessed the effects of genotype, husk-encasement status and days PSE for growing year 2009 and a second three-way ANOVA assessed the effects of genotype, husk-encasement status and growing year for silk samples harvested at 3-days PSE in both growing years. [file 12870_2019_2040_MOESM9_ESM.pdf]

Table S5. ANOVAs of the percentage of 9-monoenes relative to total alkenes.

<table>
<tr><th>Year[a]</th><th>Days PSE[b]</th><th>Two-way ANOVA[c]</th><th>Three-way ANOVA with days PSE effect[d]</th><th>Three-way ANOVA with growing year (field environment) effect[e]</th></tr>
<tr><td rowspan="2">2009</td><td>6</td><td>Genotype:
$F_{16,142}$=51.78, P<0.0001, partial R^2=0.79

Encasement status:
$F_{1,142}$=45.78, P<0.0001, partial R^2=0.04

Genotype X Encasement status:
$F_{16,142}$=1.31, P=0.1961, partial R^2=0.02

R^2=0.86</td><td rowspan="2">Genotype:
$F_{14,266}$=77.63, P<0.0001, partial R^2=0.64

Encasement status:
$F_{1,266}$=150.30, P<0.0001, partial R^2=0.09

Days PSE[b]:
$F_{1,266}$=1.82, P=0.1785, partial R^2=0.00

Genotype X Encasement status:
$F_{14,266}$=2.97, P=0.0003, partial R^2=0.02

Genotype X Days PSE:
$F_{14,266}$=8.11, P<0.0001, partial R^2=0.07

Encasement status X Days PSE:
$F_{1,266}$=5.47, P=0.0201, partial R^2=0.00

Genotype X Encasement status X Days PSE:
$F_{14,266}$=0.48, P=0.9410, partial R^2=0.00

R^2=0.84</td><td>Not applicable</td></tr>
<tr><td>3</td><td>Genotype:
$F_{15,148}$=34.63, P<0.0001, partial R^2=0.62

Encasement status:
$F_{1,148}$=125.41, P<0.0001, partial R^2=0.15

Genotype X Encasement status:
$F_{15,148}$=2.53, P=0.0023, partial R^2=0.05

R^2=0.77</td><td rowspan="2">Genotype:
$F_{6,148}$=95.28, P<0.0001, partial R^2=0.65

Encasement status:
$F_{1,148}$=47.24, P<0.0001, partial R^2=0.05

Growing year:
$F_{1,148}$=2.00, P=0.1593, partial R^2=0.00

Genotype X Encasement status:
$F_{6,148}$=4.29, P=0.0005, partial R^2=0.03

Genotype X Growing year:
$F_{6,148}$=6.34, P<0.0001, partial R^2=0.04

Encasement status X Growing year:
$F_{1,148}$=1.96, P=0.1636, partial R^2=0.00

Genotype X Encasement status X Growing year:
$F_{6,148}$=0.55, P=0.7698, partial R^2=0.00

R^2=0.83</td></tr>
<tr><td>2010</td><td>3</td><td>Genotype:
$F_{21,198}$ =15.59, P<0.0001, partial R^2=0.57

Encasement status:
$F_{1,198}$=23.51, P<0.0001, partial R^2=0.04

Genotype X Encasement status:
$F_{21,198}$=1.00, P=0.4682, partial R^2=0.04

R^2=0.65</td><td>Not applicable</td></tr>
</table>

[a]Year the inbred lines were grown; [b]Days post-silk emergence (PSE) when the silks were harvested. 3- *versus* 6-days PSE represents two different durations of exposure to the external environment; [c]Two-way full factorial analysis of variance (ANOVA) of the main effects, genotype and encasement status, and their interaction; [d]Three-way full factorial ANOVA of the main effects, genotype, encasement status, and days PSE, and all two- and three-way interactions; [e]Three-way full factorial ANOVA of the main effects, genotype, encasement status, and growing year (*i.e.* field environment), and all two- and three-way interactions; [cde]Main effects are in bold and interaction terms are in italics, F statistics and corresponding p-values are given following the main effects and interaction terms, R^2 values indicate the proportion of variance in the percentage of 9-monoenes relative to total alkenes explained by the full model and partial R^2 values indicate the proportion of variance explained by each effect.
