# Supplementary material for: Genetic and environmental variation impact the cuticular hydrocarbon metabolome on the stigmatic surfaces of maize
Source: BMC Plant Biol. 2019 Oct 17;19:430. doi: 10.1186/s12870-019-2040-3 (PMC6796380; doi:10.1186/s12870-019-2040-3)
Supplement: Supplementary file 10 — Additional file 10: Table S6. Polymorphic amino acids between B73 and Mo17 alleles of sacd genes. Sacd syntelogs in the Mo17 and B73 genomes were obtained from precomputed resources at MaizeGDB [51, 52] and amino acid polymorphisms were identified via sequence alignments. Specific single amino acid and insertion/deletion polymorphisms between the two alleles are presented. [file 12870_2019_2040_MOESM10_ESM.pdf]

Table S6. Polymorphic amino acids between B73 and Mo17 alleles of *sacd* genes.

Gene	B73 allele	Mo17 allele[a]	Single amino acid polymorphisms[b]	Insertion/deletion polymorphisms
sacd1	ACG42225.1[c]	Zm00009a016249_P003	None	None
sacd2	Zm00001d012033_P002	Zm00009a036056_P004	None	None
sacd3	ACG42431.1[c]	Zm00009a002211_P001	$D^{72}E$	$(--)^{48}AT$
sacd4	Zm00001d030328_P001	Zm00009a002343_P001	None	N-terminal 33 amino acid insertion in Mo17 allele
sacd5	Zm00001d004019_P001	Zm00009a010136_P001	None	None
sacd6	GRMZM2G027673_P03[d]	Zm00009a019762_P001	None	None
sacd7	Zm00001d014498_P001	Zm00009a023598_P001	None	None
sacd8	Zm00001d021059_P001	Zm00009a032018_P001	$H^{42}N$, $P^{442}Q$, $V^{118}A$, $R^{248}H$, $C^{286}S$	None
sacd9	Zm00001d012221_P001	Zm00009a036173_P001	$E^{294}D$	None
sacd10	Zm00001d024273_P001	Zm00009a006456_P002	**$H^{205}R$**, $G^{309}S$	None
sacd11	Zm00001d025170 _P001	Zm00009a007118_P001	$S^{352}R$	None

[a]Synteologous correspondence between Mo17 and B73 sequences were obtained from precomputed resources at MaizeGDB [51, 52].
[b]Specific amino acid polymorphisms are presented in $X^{\#}Y$ format, for which X and Y are the residues in the B73 and Mo17 alleles, respectively, and # is the position of the polymorphic residue relative to the B73 protein sequence. An amino acid polymorphism located within a conserved homodimer interface domain is boldfaced.
[c]The B73 sequence from Alexandrov *et al.* was used for analysis [53].
[d]The B73 sequence from Schnable *et al*. was used for analysis [54].
