# Supplementary material for: Genetic and environmental variation impact the cuticular hydrocarbon metabolome on the stigmatic surfaces of maize
Source: BMC Plant Biol. 2019 Oct 17;19:430. doi: 10.1186/s12870-019-2040-3 (PMC6796380; doi:10.1186/s12870-019-2040-3)
Supplement: Supplementary file 11 — Additional file 11: Figure S5. Percentage of even-numbered chain lengths relative to total hydrocarbon accumulation. Percentage of hydrocarbons having even-numbered acyl chain lengths on silks from inbred lines grown in 2010 (A) and 2009 (B) and analyzed at 3-days PSE. Inbred lines are ordered from low to high percentage for emerged silks. Asterisks indicate a significant difference between emerged and husk-encased silk means of a given inbred line (T-test; * P < 0.05, ** P < 0.001). Error bars represent ± standard error. IL14H, grown in 2010, did not accumulate observable amounts of even-numbered chain length hydrocarbons (A). HCs, hydrocarbons. [file 12870_2019_2040_MOESM11_ESM.pdf]

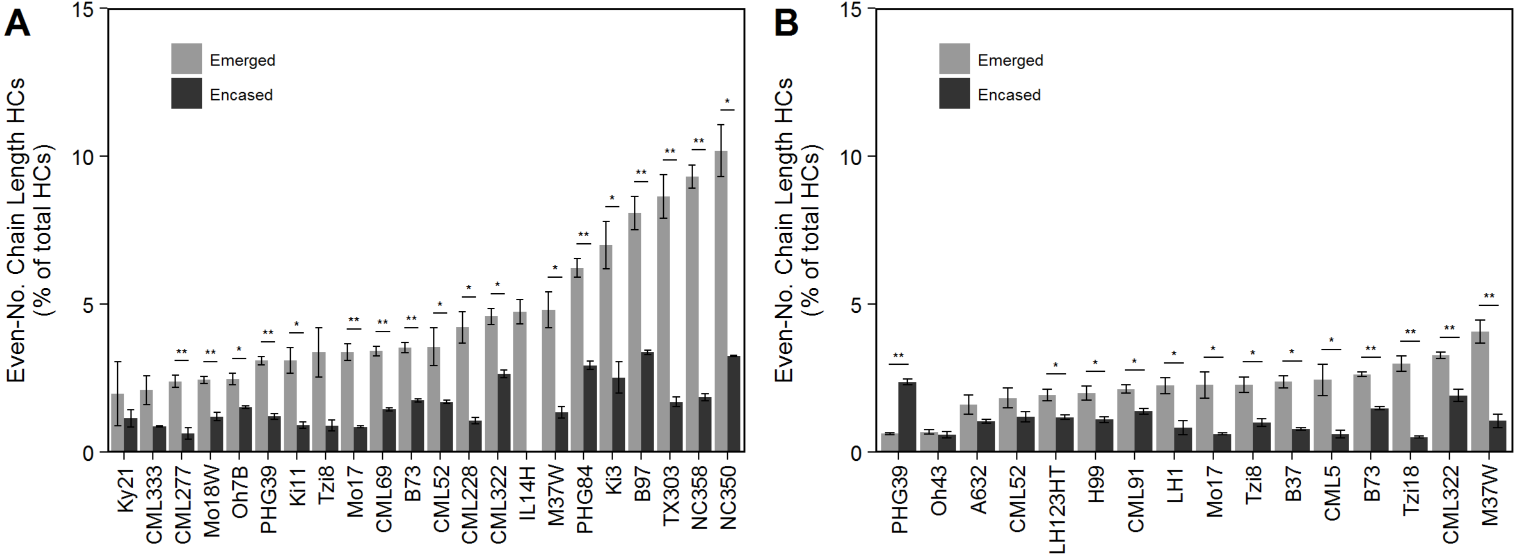

Figure S5. Percentage of even-numbered chain lengths relative to total hydrocarbon accumulation. Percentage of hydrocarbons having even-numbered acyl chain lengths on silks from inbred lines grown in 2010 **(A)** and 2009 **(B)** and analyzed at 3-days PSE**.** Inbred lines are ordered from low to high percentage for emerged silks. Asterisks indicate a significant difference between emerged and husk-encased silk means of a given inbred line (T-test; * P<0.05, ** P<0.001). Error bars represent ± standard error. IL14H, grown in 2010, did not accumulate observable amounts of even-numbered chain length hydrocarbons (**A**). HCs, hydrocarbons.
