# Supplementary material for: Genetic and environmental variation impact the cuticular hydrocarbon metabolome on the stigmatic surfaces of maize
Source: BMC Plant Biol. 2019 Oct 17;19:430. doi: 10.1186/s12870-019-2040-3 (PMC6796380; doi:10.1186/s12870-019-2040-3)
Supplement: Supplementary file 12 — Additional file 12: Table S7. ANOVAs of the percentage of even-numbered chain length hydrocarbons relative to total hydrocarbon accumulation. Two-way ANOVA assessed the effects of genotype and husk-encasement status at 3-days PSE in both growing years and at 6-days PSE in 2009. A three-way ANOVA assessed the effects of genotype, husk-encasement status and days PSE for growing year 2009 and a second three-way ANOVA assessed the effects of genotype, husk-encasement status and growing year for silk samples harvested at 3-days PSE in both growing years. [file 12870_2019_2040_MOESM12_ESM.pdf]

Table S7. ANOVAs of the percentage of even-numbered chain length hydrocarbons relative to total hydrocarbon accumulation.

<table>
<tr><th>Year[a]</th><th>Days PSE[b]</th><th>Two-way ANOVA[c]</th><th>Three-way ANOVA with days PSE effect[d]</th><th>Three-way ANOVA with growing year (field environment) effect[e]</th></tr>
<tr><td rowspan="2">2009</td><td>6</td><td>Genotype:
$F_{16,142}$=28.04, P<0.0001, partial R^2=0.25

Encasement status:
$F_{1,142}$=989.14, P<0.0001, partial R^2=0.55

Genotype X Encasement status:
$F_{16,142}$=12.33, P<0.0001, partial R^2=0.11

R^2=0.92</td><td rowspan="2">Genotype:
$F_{14,266}$=14.52, P<0.0001, partial R^2=0.11

Encasement status:
$F_{1,266}$=804.35, P<0.0001, partial R^2=0.45

Days PSE[b]:
$F_{1,266}$=48.03, P<0.0001, partial R^2=0.03

Genotype X Encasement status:
$F_{14,266}$=16.09, P<0.0001, partial R^2=0.13

Genotype X Days PSE:
$F_{14,266}$=4.47, P<0.0001, partial R^2=0.03

Encasement status X Days PSE:
$F_{1,266}$=103.87, P<0.0001, partial R^2=0.06

Genotype X Encasement status X Days PSE:
$F_{14,266}$=5.54, P<0.0001, partial R^2=0.04

R^2=0.85</td><td>Not applicable</td></tr>
<tr><td>3</td><td>Genotype:
$F_{15,148}$=8.83, P<0.0001, partial R^2=0.20

Encasement status:
$F_{1,148}$=200.54, P<0.0001, partial R^2=0.31

Genotype X Encasement status:
$F_{15,148}$=10.72, P<0.0001, partial R^2=0.25

R^2=0.77</td><td rowspan="2">Genotype:
$F_{6,148}$=14.82, P<0.0001, partial R^2=0.12

Encasement status:
$F_{1,148}$=281.73, P<0.0001, partial R^2=0.38

Growing year:
$F_{1,148}$=53.14, P<0.0001, partial R^2=0.07

Genotype X Encasement status:
$F_{6,148}$=11.93, P<0.0001, partial R^2=0.10

Genotype X Growing year:
$F_{6,148}$=0.83, P=0.5460, partial R^2=0.01

Encasement status X Growing year:
$F_{1,148}$=38.14, P<0.0001, partial R^2=0.10

Genotype X Encasement status X Growing year:
$F_{6,148}$=4.66, P=0.0002, partial R^2=0.04

R^2=0.80</td></tr>
<tr><td>2010</td><td>3</td><td>Genotype:
$F_{21,198}$ =34.75, P<0.0001, partial R^2=0.36

Encasement status:
$F_{1,198}$=788.27, P<0.0001, partial R^2=0.39

Genotype X Encasement status:
$F_{21,198}$=14.60, P<0.0001, partial R^2=0.15

R^2=0.74</td><td>Not applicable</td></tr>
</table>

[a]Year the inbred lines were grown; [b]Days post-silk emergence (PSE) when the silks were harvested. 3- *versus* 6-days PSE represents two different durations of exposure to the external environment; [c]Two-way full factorial analysis of variance (ANOVA) of the main effects, genotype and encasement status, and their interaction; [d]Three-way full factorial ANOVA of the main effects, genotype, encasement status, and days PSE, and all two- and three-way interactions; [e]Three-way full factorial ANOVA of the main effects, genotype, encasement status, and growing year (*i.e.* field environment), and all two- and three-way interactions; [cde]Main effects are in bold and interaction terms are in italics, F statistics and corresponding p-values are given following the main effects and interaction terms, R^2 values indicate the proportion of variance in the percentage of even-numbered chain length hydrocarbons relative to total hydrocarbon accumulation explained by the full model and partial R^2 values indicate the proportion of variance explained by each effect.
